# Effect and mechanisms of kaempferol against endometriosis based on network pharmacology and in vitro experiments

**DOI:** 10.1186/s12906-022-03729-4

**Published:** 2022-10-02

**Authors:** Junde Zhao, Juntao Wang, Jinxing Liu, Shuling Li, Pengfei Liu, Xiaodan Zhang

**Affiliations:** 1 Department of Traditional Chinese Medicine, Qilu Hospital of Shandong University, Jinan, 250012 China; 2 Laboratory of Basic Medical Sciences, Qilu Hospital of Shandong University, Jinan, 250012 China; 3 First College of Clinical Medicine, Shandong University of Traditional Chinese Medicine, Jinan, 250011 China; 4 Division of Hand and Foot Surgery, Department of Orthopedic Surgery, Qilu Hospital of Shandong University, Jinan, 250012 China

**Keywords:** Endometriosis, Kaempferol, Network pharmacology, Migration and invasion

## Abstract

Endometriosis is a common gynecological disease, and its underlying mechanisms remain elusive. Patients are at a higher risk of recurrence after surgery or drug withdrawal. In this study, to identify a potentially effective and safe therapy for endometriosis, we screened potential target genes of kaempferol on endometriosis using network pharmacology and further validation. Network pharmacology showed kaempferol may suppress migratory and invasive properties by modulating the phosphoinositide 3-kinase (PI3K) pathway and its downstream target matrix metalloproteinase (MMP)9. Furthermore, in vitro experiments showed that kaempferol repressed the migration and invasion of endometrial cells, and this effect may be involved in mediating the PI3K-related genes, phosphatase and tensin homolog (PTEN) and MMP9. Network pharmacology and in vitro experiments showed that kaempferol, repressed the implantation of endometrial cells and formation of ectopic lesions by inhibiting migration and invasion and regulating PTEN and MMP9, which may be associated with the PI3K pathway.

**Supplementary Information:**

The online version contains supplementary material available at 10.1186/s12906-022-03729-4.

## Introduction

Endometriosis is a common gynecological disease that affects more than 5% of women during the reproductive period [1–3]. Regardless of being benign, endometriosis has some malignant features, including migration, invasion, and recurrence. The usual clinical characteristics of endometriosis are dysmenorrhea, which gradually worsens, dyspareunia, irregular menstruation, and infertility. This dramatically affects women’s daily lives. The underlying mechanisms of endometriosis remain unclear. Endometriosis is currently treated with surgery and hormone therapy. However, hormone therapies have some side effects such as anovulation and menstrual irregularities. Furthermore, endometriosis is vulnerable to recurrence (approximately 40%) within five years of drug withdrawal, even after surgery [2, 3]. According to the retrograde menstruation theory [1], the endometrium can invade and implant ectopic tissues other than uterus. Migration and invasion play an essential role in the occurrence and development of ectopic lesions, and drugs that alleviate migration and invasion are urgently required [4]. In this study, we introduced kaempferol in the treatment of endometriosis. Using network pharmacology analysis, we predicted that kaempferol could inhibit the migration and invasion of endometriosis, and verified the efficacy of kaempferol in the treatment of endometriosis through experimental validation in vitro.

**Fig. 1:**
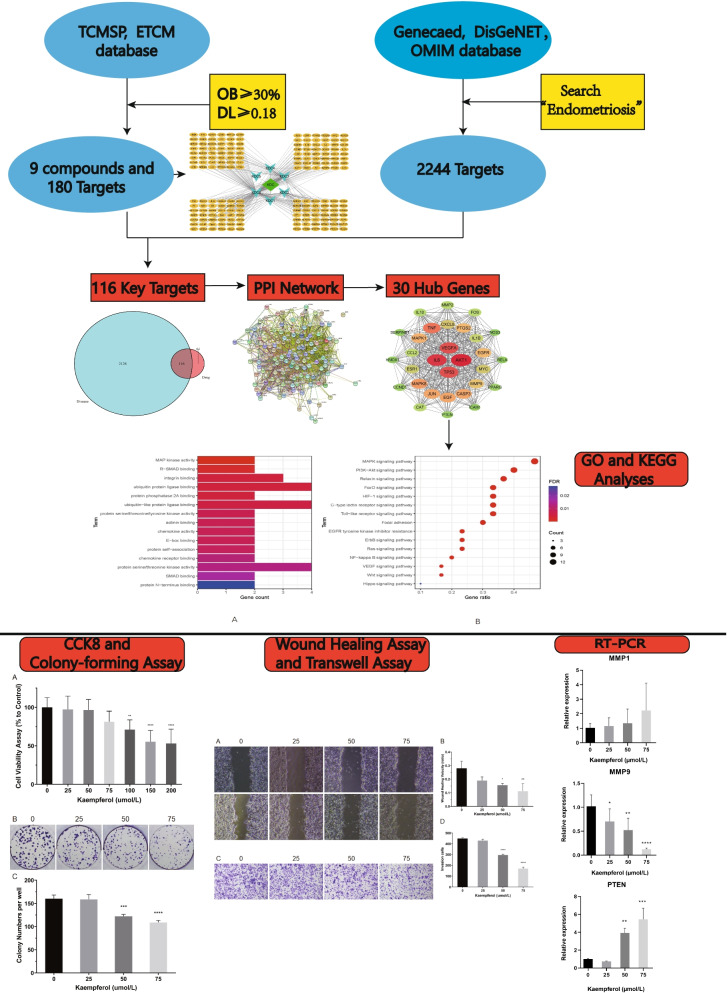
The flow chart of the study

Natural products and biomolecules have health-promoting benefits against a much wider range of ailments [5–7]. Recently, an increasing number of studies have focused on the anti-tumor effect of kaempferol and it has been widely applied in various cancers, such as pancreatic, stomach, oral, liver, kidney, and brain tumors [8–11]. Kaempferol suppresses the growth and development of cancer by regulating apoptosis, migration, and invasion via various signaling pathways [8–14]. For example, kaempferol inhibits migration and invasion by downregulating the extracellular regulating kinase (ERK)1/2 signaling pathway and matrix metalloproteinase-2(MMP2) expression in oral cancers [10]. In hormone-related cancers, such as breast cancer, kaempferol represses the expression of ERα [15]. Endometrium, a hormone-dependent condition, also possesses potential migratory and invasive traits. We hypothesized that kaempferol may be a potential therapeutic agent for endometriosis. We found that kaempferol can modulate integrin binding, ubiquitin, and ubiquitin-like protein ligase binding through the phosphoinositide 3-kinase (PI3K) pathway [16, 17], as predicted by network pharmacology analysis. In this study, we tested whether kaempferol suppressed the migration and invasion of endometriosis and paved the way for therapy with kaempferol in the future. Figure 1 showed the complete flow chart of this study.

## Materials and methods

### Prediction of targets of kaempferol

The kaempferol was analyzed using the TCMSP database (https://old.tcmsp-e.com/tcmspsearch.php?qs=molecule_name&q=kaempferol&token=d49dd3dbca4e2d3b947698f09744e051) to identify the known drug targets. And the two-dimensional structure of the kaempferol obtained from PubChem was imported into the SwissTargetPrecision database (http://www.swisstargetprediction.ch/), and a threshold (probability > 0.6) was set to obtain a possible target for each combination.

### Prediction of endometriosis targets

The keyword “endometriosis” was searched in three different disease gene databases: (1) GeneCards Human Gene database (https://www.genecards.org/Search/Keyword?queryString=endometriosis); (2) DisGeNET database (https://www.disgenet.org/browser/0/1/0/C0014175/); (3) Online Mendelian Inheritance in Man (OMIM) database (https://omim.org/search?index=entry&start=1&limit=10&sort=score+desc%2C+prefix_sort+desc&search=endometriosis). UniProt (http://www.UniProt.org) was used to obtain the gene symbols of all targets (endometriosis and kaempferol), and the information was used for subsequent network pharmacological data analysis. (4) Datasets contain endometriosis tissue and normal endometrial tissue sequencing results from GEO database (http://www.ncbi.nih.gov/geo) to download. The raw data were downloaded as MINiML files. Using the limma package in the R software to study the differentially expressed mRNA. The adjusted *P*-value was analyzed to correct the false positive results in GEO datasets. “Adjusted *P* < 0.05 and Log (Fold Change) >1 or Log (Fold Change)<−1” were defined as the threshold for the differential expression of mRNAs.

### Screening for key targets

The Venny R package was employed to map the targets of kaempferol and the known therapeutic targets of endometriosis to build a Venn diagram. We combined the crossed targets of kaempferol and endometriosis after deleting the duplicate targets of endometriosis. These cross targets were defined as critical targets for the treatment of endometriosis.

We used the Cytoscape software (version 3.7.2, Boston, MA, USA) to construct the drug-ingredient-target interaction network.

### Protein-Protein Interaction (PPI) network construction

The key targets were subjected to PPI analysis using the STRING database (https://string-db.org/), and the species “*Homo sapiens*” was selected to generate a PPI network. After processing the data by R software (R version 4.1.2), setting the minimum required interaction score highest confidence (0.900) ,we obtained the top 30 hub genes. Subsequently, we imported the hub gene data into the Cy software and obtained the HUB gene PPI network diagram.

### Gene Ontology (GO) and Kyoto Encyclopedia of Geld genomes (KEGG) analyses of hub genes

We imported 30 critical genes into the Bioconductor library of R software to perform GO and KEGG pathway enrichment analyses. The screening criteria were set at *P*<0.05, *Q*<0.05, and the restricted species was *Homo sapiens*. GO and KEGG enrichment analyses revealed the top 15 results. Bar and bubble graphs represent the visualization, and a pathway atlas is presented for related pathways that meet clinical significance.

### Cell culture

The Ishikawa cells were used in this study. The Ishikawa cell line was obtained from the Basic Medical Laboratory of Qilu Hospital. The cells were cultured in DMEM (Life Technologies, USA) containing 1% penicillin/streptomycin (Gibco, USA) and 10% FBS (Gibco, USA) in a humidified atmosphere containing 5% CO_2_ at 37 °C. The cells were passaged when they reached 85% confluence and stored in liquid nitrogen for long-term preservation.

### Cell viability assay

Kaempferol was purchased from Solarbio Science &Technology Co.,Ltd (Beijing, China) [18]. The purity of kaempferol used in the following experiments was>98%. Kaempferol was dissolved in DMSO at a concentration of 200 mmol/L.

Ishikawa cells were seeded in 96-well plates at a density of 5,000 cells per well and cultured in a complete cell culture medium for 24 h for recovery. Next, the medium was changed to DMEM with 0, 25, 50, 75, 100, 150, and 200 µmol/L kaempferol, without FBS, and cultured for 48 h. Then, 10 μL of sterile Cell Counting Kit (CCK)8 solution (Bimake, USA) was added to each well and incubated for another 1.5 h at 37 °C. Absorbance was measured at 450 nm using a microplate reader.

### Colony-forming assay

Ishikawa cells were seeded at a density of 500 cells per well in a six-well plate and cultured in a complete cell culture medium for 6 h for recovery [19]. Kaempferol was added to the medium at different concentrations, with end densities of 0, 25, 50, and 75 µmol/L. The cells were cultured for a week, and then the medium was gently removed, fixed with 4% paraformaldehyde for 10 min, stained with crystal violet for 15 min, washed, and observed under a microscope.

### Wound healing test

Ishikawa cells were seeded at a density of 10, 000 cells per well in a six-well plate and cultured in a complete medium until they reached 85% confluence [20]. Then, a scratch was made to cause a wound, the cells were gently washed, and the remaining cells were incubated with a medium containing DMEM and 0, 25, 50, and 75 µmol/L kaempferol without FBS. The wound surface areas were measured for 24 h to evaluate the ability of the cells to migrate.

### Transwell test

The transwell plates were coated with gelatin and cultured in a complete medium for 6 h to recover [21]. Ishikawa cells were diluted with serum-free DMEM containing 0, 25, 50, and 75 µmol/L kaempferol, and inoculated into the upper well of the transwell plate at a density of 100,000 cells per well. The lower well was DMEM complete medium containing 20%FBS to aid their migration. The cells were then incubated in a cell culture container for 24 h. Then, the cells were fixed with 4% paraformaldehyde for 10 min, and the cells in the upper side of the wells were scratched gently; the cells on the lower side were stained with crystal violet and counted under a microscope.

### Real-time RT-PCR

Ishikawa cells were seeded at a density of 10,000 cells per well in a six-well plate overnight. Afterward, the cells were incubated with the medium containing DMEM and 0, 25, 50, and 75 µmol/L kaempferol [22]. Total RNA was collected using a RNeasy mini kit (RNA Fast 200, China), according to the manufacturer’s instructions. Then, cDNA was synthesized from 1 μg of total RNA using a SureScript First-Strand cDNA Synthesis Kit (GeneCopoeia, USA). Finally, the expression levels of GAPDH, PTEN, MMP1, and MMP9 were measured using an all-in-one qRT-PCR detection kit (GeneCopoeia, USA). The sequences of the primers were as follows: PTEN, 5′-CAAGATGATGTTTGAAACTATTCCAATG-3′ (sense) and 5′-CCTTTAGCTGGCAGACCACAA-3′ (antisense); MMP1, 5′-CACAAACCCCAAAAGCGTGT-3′ (sense) and 5′-TCGGCAAATTCGTAAGCAGC-3′ (antisense); MMP9, 5′-TCTGCCCCGGACCAAGGATA-3ʹ (sense) and 5′-ACATAGGGTACATGAGCGCC-3′ (antisense); and GADPH, 5′-GCACCGTCAAGGCTGAGAAC-3′ (sense) and 5′-TGGTGAAGACGCCAGTGGA-3′ (antisense). Melting curve analysis was used to confirm the amplification specificity. The quantification data were analyzed using LightCycler analysis software (version 4.0; Roche Applied Science, Mannheim, Germany). Relative expression was normalized to that of GAPDH.

### Western blot assay

Ishikawa cells were seeded at a density of 10,000 cells per well in a six-well plate overnight. Afterward, the cells were incubated with the medium containing DMEM and 0, 25, 50, and 75 µmol/L kaempferol for 24 hours. Total proteins were extracted with RIPA buffer (Solarbio, #R0020) which added protease inhibitors (Solarbio, #P0100) and phosphatase inhibitors (Solarbio, #P1260). The lysates were separated on SDS-PAGE gels under 80V. After that, the proteins were immediately trans-blotted to a Millipore PVDF membrane (0.2 µm) under constant current. The blot was blocked with a protein-free rapid blocker(Epizyme,#PS108P) at room temperature for 1 h, and then incubated overnight with primary antibody(abcam, #ab267787;Huabio, # HA721136) at 4℃.Next day, the blots were washed with TBST for 3 times and incubated with secondary antibodies for 1 h. The blots were detected by ECL reagent (Millipore) using a Tanon System for visualization after washing with TBST for 3 times. GAPDH expression was used to quantify the data. ImageJ(2.3.0) was used to analysis (version 5.2.1).

### Statistical analysis

SPSS 22.0 was used to analyze the data. Quantitative data are presented as the mean ± SD of at least three independent experiments. One-way ANOVA were employed to compare the means. Differences were considered statistically significant if the *p-value* was <0.05 or <0.01.

## Results

### The candidate targets of kaempferol

According to the TCMSP, and SwissTargetPrecision databases, we identified a total of 148 potential targets after deduplication.

### Prediction of therapeutic targets of endometriosis

**Fig. 2:**
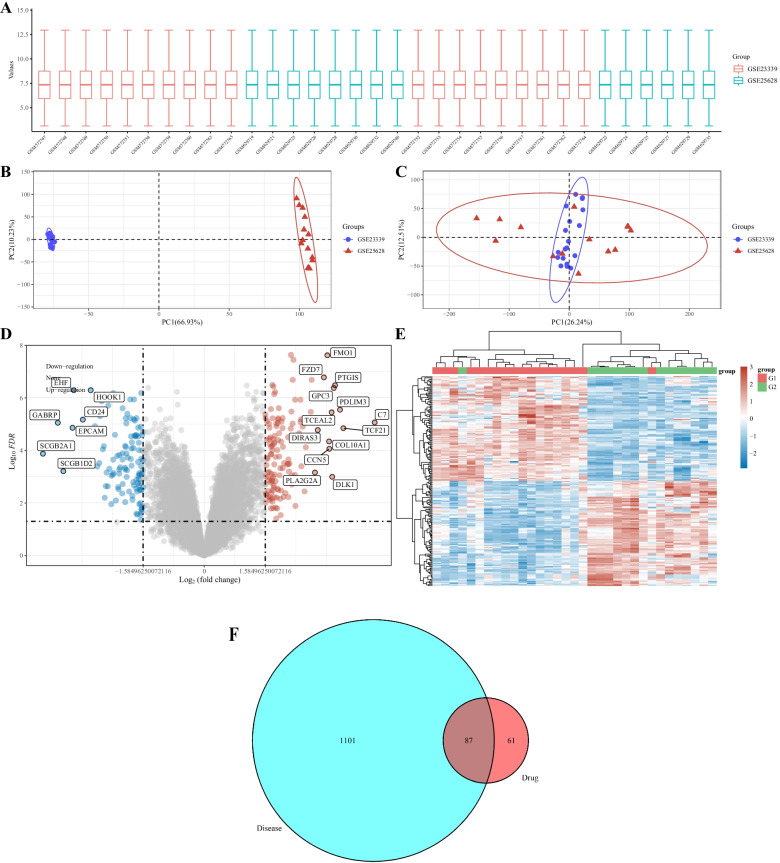
**A** The boxplot of the normalized data. Different colors represent different datasets. Rows represent samples, and columns represent the gene expression values in the samples. **B** PCA results before batch removal for multiple datasets. Different colors represent different datasets. As shown in the schematic diagram, three datasets are separated without any intersection. **C** PCA results after batch removal, as shown in the schematic diagram shows the intersection of three datasets, which can be used in subsequent analysis. **D** Volcano plot:The volcano plot was constructed using the fold change values and P-adjust. Red dots indicate upregulated genes; blue dots indicate downregulated genes. **E** The heatmap of the differential gene expression, different colors represent the trend of gene expression in different tissues. The top 50 up-regulated genes and top 50 down-regulated genes were showed in this figure. **F** Venn diagram of targets of active ingredients of kaempferol and those related to endometriosis

We searched the GeneCards Human Gene Database, DisGeNET database, and Online Mendelian Inheritance in Man database for “endometriosis”. In addition, the differentially expressed genes in endometriosis compared with normal tissues were analyzed from two data sets GSE23339 and GSE25628 in GEO database (Fig. 2 A-E). A total of 1188 targets were identified after deduplication.

### Screening for key targets

Using the Venny R package, we obtained 87 key targets to establish a Venn diagram (Fig. 2F).

### Construction of the PPI network and selection of HUB genes

**Fig. 3:**
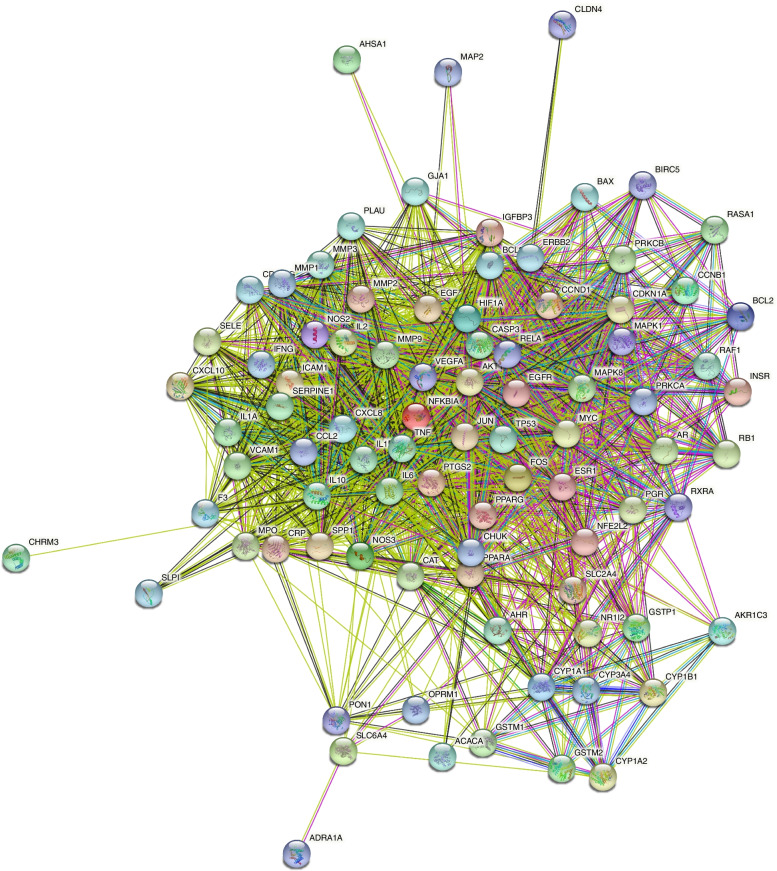
Protein-protein interaction (PPI) network of the common targets of active ingredients of kaempferol and endometriosis

**Fig. 4:**
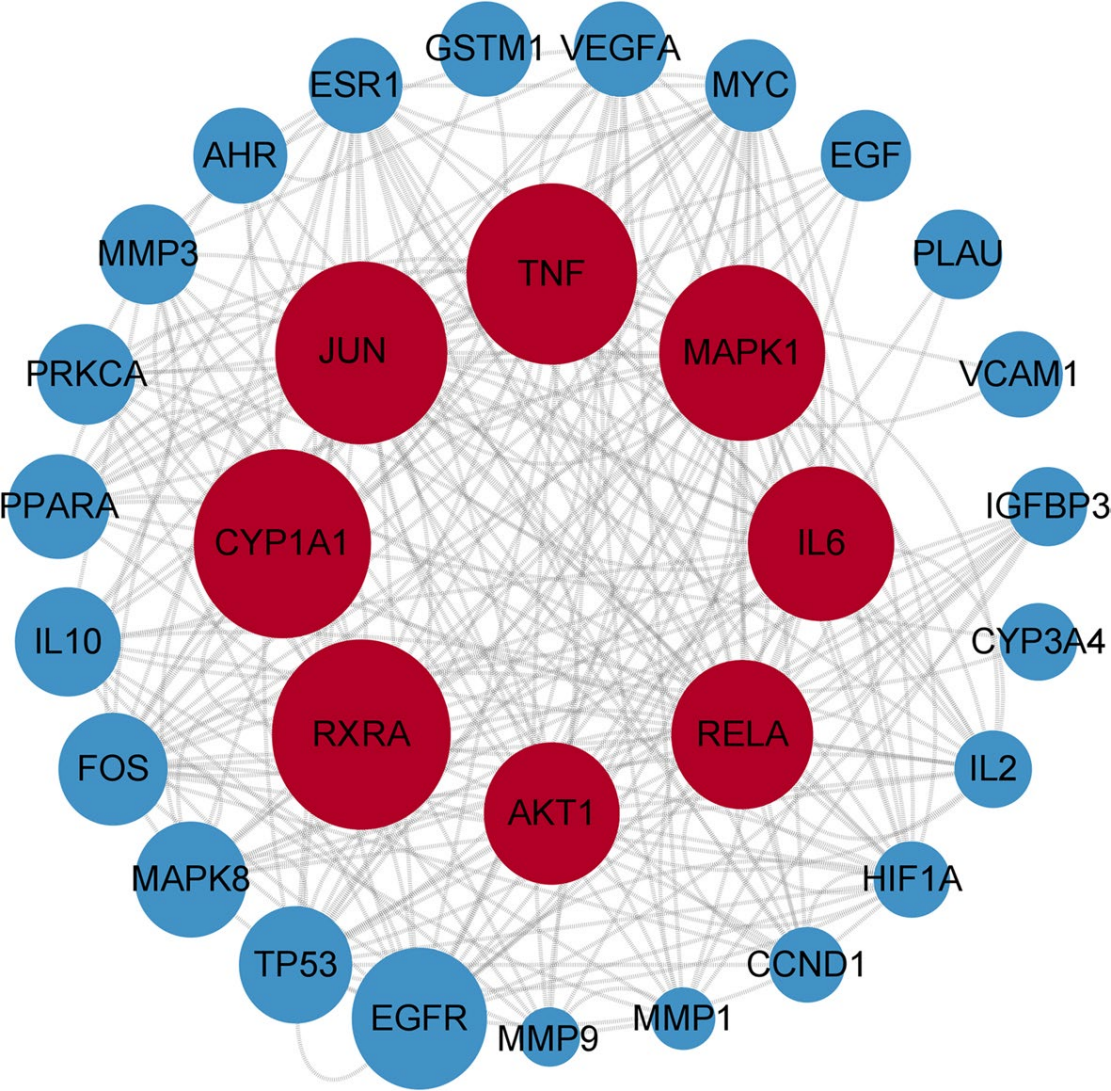
Hub gene interactions in the protein-protein interaction (PPI) network: 30 significant hubs according to the degree value; from large to small

As depicted in Fig. 3, we obtained the PPI network using the STRING database and the restricted species Homo sapiens. We obtained 30 hub genes through PPI results and the Cytoscape plug-in cytoHubba (Fig. 4).

### GO and KEGG pathway enrichment analyses

We imported 30 critical genes into the Bioconductor library of the R software for GO and KEGG pathway enrichment analyses. The top 15 results and bar and bubble graphs were obtained.

**Fig. 5:**
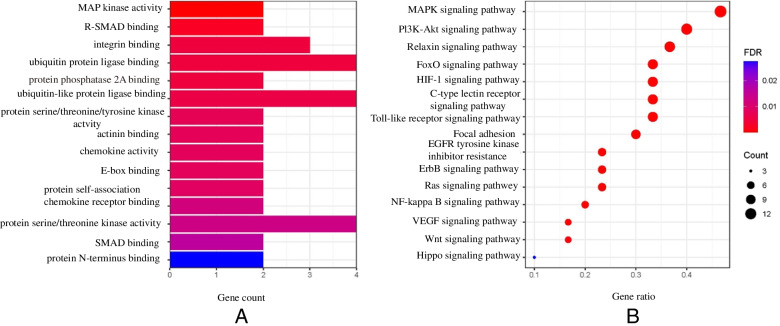
Bar graphs showing GO analyses of the 15 hub genes (**A**). Bubble graphs showing KEGG analyses of the 15 hub genes (**B**)

GO analysis revealed that mitogen-activated protein kinase (MAPK) activity, R-(mothers against decapentaplegic) SMAD binding, integrin binding, ubiquitin, and ubiquitin-like protein ligase binding may be vital biological processes related to the treatment of endometriosis by kudingcha. KEGG analysis showed that the MAPK, PI3K-Akt, Forkhead box O (FoxO), and HIF-1 signaling pathways might be the central pathways associated with the treatment of endometriosis by kaempferol (Fig. 5).

### Kaempferol impairs cell viability and proliferation of Ishikawa cells in a concentration-dependent pattern

**Fig. 6:**
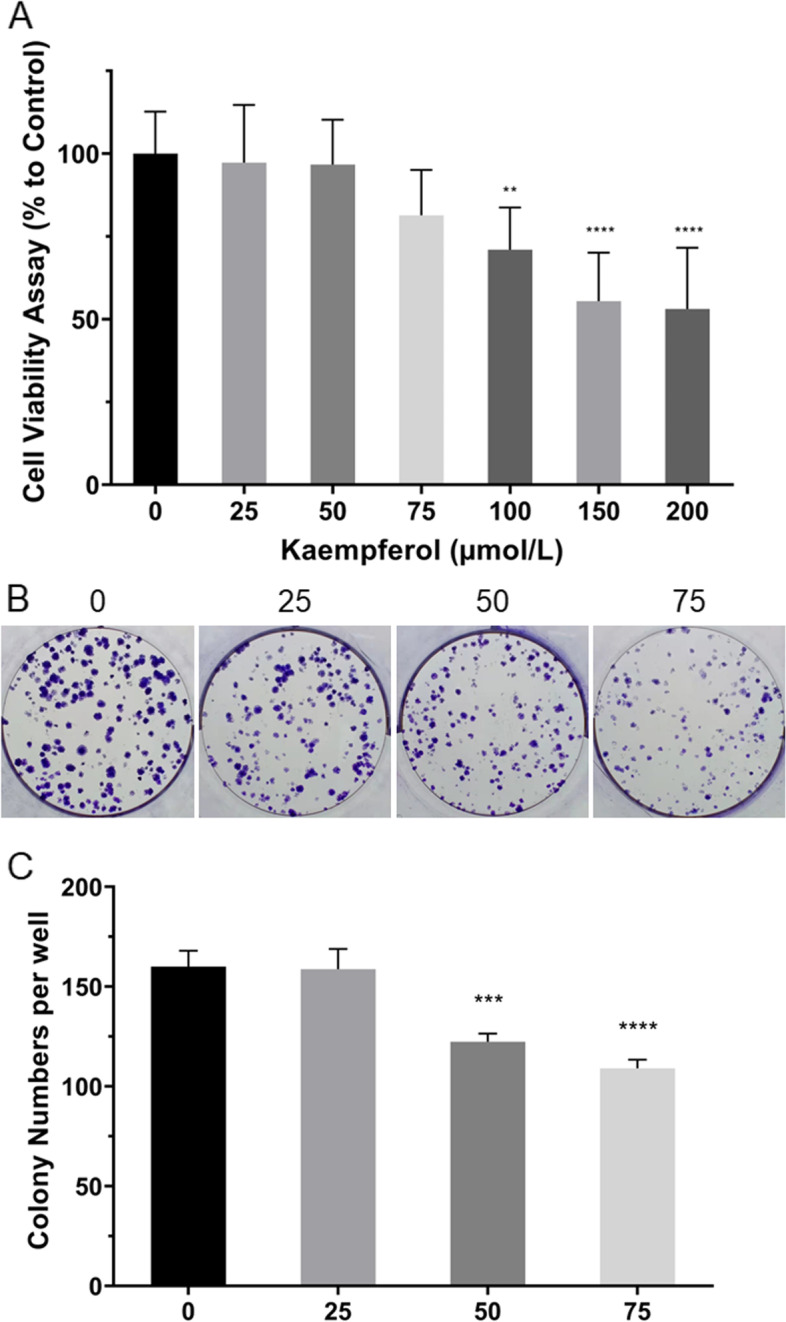
Effect of kaempferol on the viability and proliferation of Ishikawa cells. **A** CCK8 assay showed that kaempferol did not affect the viability of Ishikawa cells at low concentrations (25, 50, and 75 µmol/L) while reduced the viability of endo cells in a concentration-dependent pattern at high concentrations (100, 150, and 200 µmol/L). **B** and (**C**) Colony-forming assay showed that kaempferol did not affect the proliferation of Ishikawa cells at 25 µmol/L but had an inhibitory effect at 50 and 75 µmol/L. ** *P*<0.01, ***P<0.001, **** *P*<0.0001

To evaluate the effect of kaempferol on cell viability, Ishikawa cells were treated with different concentrations of kaempferol for 48 h. Then, the CCK8 assay was used to assess cell viability. The results showed that at low concentrations of 25, 50, and 75 µmol/L, kaempferol did not affect the viability of Ishikawa cells (*P*>0.05); however, at high concentrations of 100, 150, and 200 µmol/L, kaempferol decreased the viability of Ishikawa cells (*P*<0.01) (Fig. 6A). The effect of kaempferol on the proliferation of Ishikawa cells was evaluated via a colony-forming assay. The results showed no significant difference in colony numbers between 0, and 25 µmol/L kaempferol, whereas the number of cell colonies decreased significantly at 50, and 75 µmol/L kaempferol compared to 0 µmol/L (*P*<0.01) (Fig. 6B and C).

### Kaempferol decreases cell migration and invasion of Ishikawa cells

**Fig. 7:**
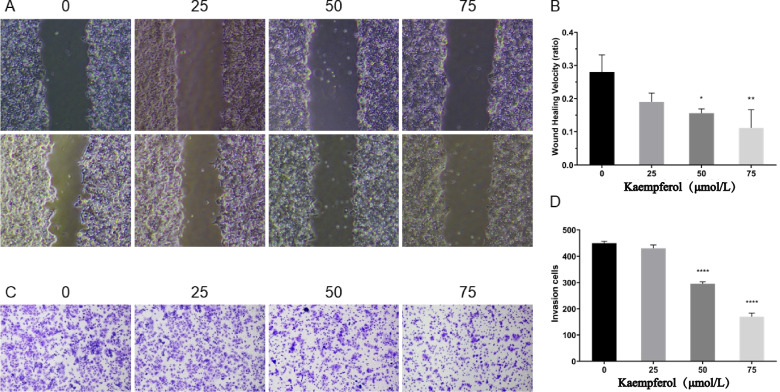
Effect of kaempferol on the migration and invasion of Ishikawa cells. **A** and **B** show that kaempferol inhibits the migration of Ishikawa cells in a wound healing assay; **C** and **D** show that kaempferol impairs the invasion ability of Ishikawa cells in a transwell assay. ** *P*<0.01, ***P<0.001, **** *P*<0.0001

A wound healing assay was performed to evaluate the effect of kaempferol on the migration of Ishikawa cells. The results showed no significant difference in the migration distance between the 0 and 25 µmol/L kaempferol treatment groups (*P*>0.05); however, the migration distance was significantly decreased by 50 and 75 µmol/L kaempferol (*P*<0.05, or *P*<0.01) (Fig. 7A and B). Cell invasion ability was evaluated via a Transwell assay. The results showed no significant difference in the number of cells on the lower surface between the 0 and 25 µmol/L kaempferol groups; in contrast, the numbers were significantly decreased in the 50 and 75 µmol/L kaempferol-treated groups compared to the control group (*P*<0.01) (Fig. 7C and D).

### Kaempferol upregulated the expression of PTEN while downregulated that of MMP9 in Ishikawa cells

**Fig. 8:**
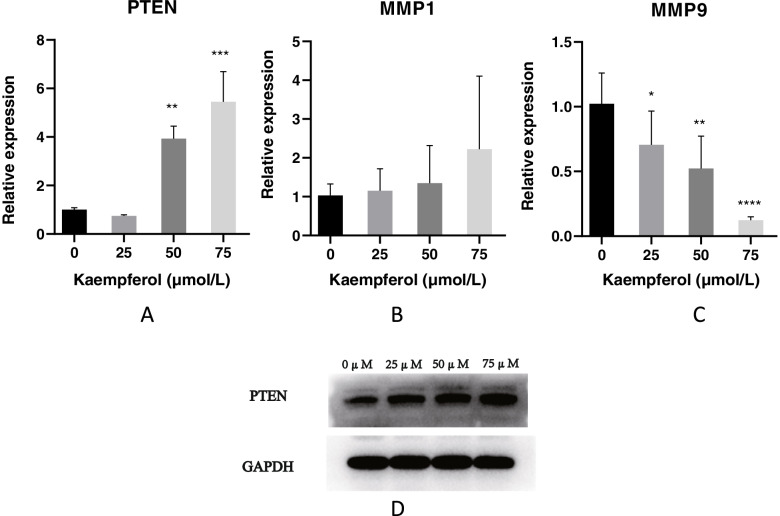
Effect of kaempferol on the predicted genes associated with migration and invasion of Ishikawa cells. **A** Kaempferol significantly upregulated the expression of PTEN in endometrial cells; **B** Kaempferol did not significantly affect MMP1 levels in endometrial cells; **C** Kaempferol significantly reduced MMP9 levels in endometrial cells; **D** Kaempferol enhanced the expression of PTEN in endometrial cells by western blotting. *P<0.05, ** *P*<0.01, ***P<0.001, **** *P*<0.0001

Real-time RT-PCR was performed to determine the potential mechanism of action of kaempferol in endometriosis. The results demonstrated that expression of PTEN was attenuated after treatment with 50 and 75 µmol/L kaempferol (Fig. 8A). At the same time, 25 µmol/L kaempferol treatment did not result in differential alterations compared with the control group. In addition, MMP9 expression was significantly inhibited after treatment with 25, 50, and 75 µmol/L kaempferol (Fig. 8C). However, there was no significant difference in MMP1 expression after treatment with kaempferol (Fig. 8B). We further performed Western blot assay on the changes of PTEN. The results showed that the protein expression level of PTEN increased with kaempferol concentration (Fig. 8D).

## Discussion

Traditional Chinese medicine has been used to treat various diseases for thousands of years and has achieved considerable accomplishments. However, the mechanisms remain ambiguous because herbs exert multifaceted effects through multiple pathways. The main ingredients and target genes can be identified by network analysis. In this study, we aimed to identify the primary target genes of kaempferol involved in the treatment of endometriosis. Using GO analysis, we found that MAP kinase activity, R-SMAD binding, integrin binding, ubiquitin, and ubiquitin-like protein ligase binding may be vital biological processes affected by kaempferol. In addition, KEGG analysis revealed that the MAPK, PI3K-Akt, FoxO, and Hypoxia-inducible factors (HIF)-1 signaling pathways may be the central signaling pathways associated with the treatment of endometriosis by kaempferol. Subsequently, we assessed the migration and invasion of endometrial cells. In addition, the expression of PTEN and MMPs, which are target genes associated with the PI3K/Akt/MMP signaling pathway, was evaluated by real-time PCR, considering the data mining results. The experiments suggested that kaempferol, may inhibit the migration and invasion of endometrial cells by regulating the PI3K/Akt/MMP signaling pathway, coinciding with predictions by network pharmacology and data mining.

Endometriosis is a common gynecological disease. Because of the migration and invasion of epithelial cells, the endometrium can develop ectopic lesions in organs other than the uterus. Kaempferol has been shown to suppress migration and invasion in many cancers [5, 13, 14, 23]. For instance, kaempferol inhibits the migration and invasion of human retinal pigment epithelial cells [24]. Recent studies have shown that kaempferol represses migration and invasion via various signaling pathways. Kaempferol regulates PI3K/ mammalian target of rapamycin (mTOR)/MMPs protein pathways in liver cancers [25]. Moreover, kaempferol blocks the MAPK signaling pathway and downstream MMP expression to influence migration and invasion in rheumatoid arthritis [26]. In this study, we used the Cytoscape software to identify the main target of kaempferol via drug-ingredient-target interaction network and GO and KEGG analyses. The results demonstrated that kaempferol might alleviate migration and invasion in endometriosis via the PI3K signaling pathway and downstream MMP expression. The subsequent experiments involving wound healing and transwell assays showed that kaempferol reduced migration and invasion without affecting cell proliferation, which was in accordance with the prediction. Furthermore, RT-PCR was used to quantify the genes associated with the signaling pathways. The results showed that kaempferol increased the expression of PTENs, which are related to the PI3K pathway, and decreased the expression of MMP9.

The PI3K/AKT/mTOR pathway participates in various processes including proliferation, migration, invasion, and apoptosis. Several studies have shown that the inactivation of this pathway could prevent the recurrence of endometriosis [27]. And the results of KEGG analysis also identified that the effect of kaempferol on endometriosis may be related to PI3K/AKT/mTOR pathway [28–30]. The downstream gene, PTEN, is downregulated in both eutopic and ectopic endometrium from patients with endometriosis [31, 32]. Additionally, PTEN and platelet-derived growth factor (PDGF) play a role in the migration and invasion of endothelial cells and enhance the formation of ectopic lesions [33]. Migration and invasion are vital processes in the development and metastasis of cancer [34, 35]. The MMPs are central to many pathophysiological activities such as apoptosis, migration, and growth [36]. Among MMPs, MMP2 has been shown to mediate migration aninvasion in gliomas through focal adhesion kinase (FAK) and Janus kinase (JAK)-signal transducer and activator of transcription (STAT) signaling [37, 38]. Additionally, MMP1 and MMP14 are associated with migration and invasion [34, 35]. As far as endometriosis is concerned, increased expression of MMPs has been used as a biomarker in endometriosis [39]. Additionally, the PI3K pathway and MMP9 participate in modulating the migration and invasion of endometrial cells [40]. In this study, the results of wound healing and transwell assays showed that kaempferol inhibited the migration and invasion of endometrial cells. Kaempferol attenuated the expression of PTEN and led to downregulation of MMP9, as suggested by RT-PCR results. These results indicated that kaempferol suppressed the migration and invasion of endothelial cells by modulating the expression of genes associated with the PI3K pathway.

However, this study has several limitations. First, the analysis was performed based on TCMSP, ETCM, and SwissTargetPrecision databases. This might have led to biased results. Second, data mining analysis is a prediction and requires further validation. We validated the selected genes and pathways in addition to this research. This may be omitted.

In conclusion, kaempferol plays vital roles in the migration and invasion of endometriosis, as indicated by network pharmacology and data mining. Experiments validated the protective effect of kaempferol, and the potential impact that may be involved in the regulation of PI3K/MMPs.

## Supplementary Information

**Additional file 1.**

## Data Availability

The data in the current study come from TCMSP database (https://old.tcmsp-e.com/tcmsp.php) and STRING database (https://string-db.org/). All data generated or analyzed in this study are included in this article and its additional files.

## Abbreviations

PI3K: Phosphoinositide 3-kinase
MMP: Matrix metalloproteinase
PTEN: Phosphatase and tensin homolog
ERK1/2: Extracellular regulating kinase1/2
TCMSP: Chinese Medicine Systems Pharmacology
ETCM: Encyclopedia of Traditional Chinese Medicine
OB: Oral bioavailability
DL: Drug-likeness
OMIM: Online Mendelian Inheritance in Man
PPI: Protein-protein interaction
GO: Gene Ontology
KEGG: Kyoto Encyclopedia of Geld genomes
CCK: Cell Counting Kit
MAPK: Mitogen-activated protein kinase activity
R-SMAD: R-mothers against decapentaplegic
FoxO: Forkhead box O
HIF-1: Hypoxia-inducible factors-1
mTOR: mammalian target of rapamycin
PDGF: Platelet-derived growth factor
FAK: Focal adhesion kinase
JAK: Janus kinase
STAT: Signal transducer and activator of transcription
