# Supplementary material for: Effect and mechanisms of kaempferol against endometriosis based on network pharmacology and in vitro experiments
Source: BMC Complement Med Ther. 2022 Oct 2;22:254. doi: 10.1186/s12906-022-03729-4 (PMC9528065; doi:10.1186/s12906-022-03729-4)
Supplement: Supplementary file 1 — Additional file 1. [file 12906_2022_3729_MOESM1_ESM.zip › code for KEGG.docx]

install.packages("colorspace")

install.packages("stringi")

source("http://bioconductor.org/biocLite.R")

biocLite("DOSE")

biocLite("clusterProfiler")

biocLite("pathview")

setwd("C:\\Users ")

library("clusterProfiler")

rt=read.table("id.txt",sep="\t",header=T,check.names=F)

rt=rt[is.na(rt[,"entrezID"])==F,]

geneFC=rt$logFC

gene=rt$entrezID

names(geneFC)=gene

Enrichment Analysis

kk<-enrichKEGG(gene=gene,organism="hsa",pvalueCutoff=0.01,qvalueCutoff=0.01)

write.table(kk,file="KEGG.txt",sep="\t",quote=F,row.names=F)

Histogram

tiff(file="barplot.tiff",width=35,height=20,units="cm",compression="lzw",bg="white",res=600)

barplot(kk,drop=TRUE,showCategory=20)

dev.off()

Dotplot

tiff(file="dotplot.tiff",width=35,height=20,units="cm",compression="lzw",bg="white",res=600)

dotplot(kk,showCategory=20)

dev.off()

Pathway map

library("pathview")

keggxls=read.table("KEGG.txt",sep="\t",header=T)

for(i in keggxls$ID){

pv.out<-pathview(gene.data=geneFC,pathway.id=i,species="hsa",out.suffix="pathview")

}
