# Supplementary material for: Effect and mechanisms of kaempferol against endometriosis based on network pharmacology and in vitro experiments
Source: BMC Complement Med Ther. 2022 Oct 2;22:254. doi: 10.1186/s12906-022-03729-4 (PMC9528065; doi:10.1186/s12906-022-03729-4)
Supplement: Supplementary file 1 — Additional file 1. [file 12906_2022_3729_MOESM1_ESM.zip › Drug -EMS.docx]

PGR

PTGS2

CHRM3

ADRA1A

SLC6A4

OPRM1

BCL2

BAX

JUN

CASP3

PRKCA

PON1

MAP2

NOS2

AR

RELA

AKT1

TNF

AHSA1

MAPK8

MMP1

PPARG

CYP3A4

CYP1A2

CYP1A1

ICAM1

SELE

VCAM1

NR1I2

CYP1B1

GSTP1

AHR

SLC2A4

INSR

GSTM1

GSTM2

AKR1C3

SLPI

ESR1

RXRA

CAT

MMP3

EGFR

VEGFA

CCND1

BCL2L1

FOS

CDKN1A

PLAU

MMP2

MMP9

MAPK1

IL10

EGF

RB1

IL6

TP53

NFKBIA

RAF1

HIF1A

ERBB2

ACACA

MYC

F3

GJA1

IL1B

CCL2

CXCL8

PRKCB

BIRC5

NOS3

IL2

CCNB1

SERPINE1

IFNG

IL1A

MPO

NFE2L2

CLDN4

PPARA

CRP

CXCL10

CHUK

SPP1

IGFBP3

CD40LG

RASA1
