# Supplementary material for: Effect and mechanisms of kaempferol against endometriosis based on network pharmacology and in vitro experiments
Source: BMC Complement Med Ther. 2022 Oct 2;22:254. doi: 10.1186/s12906-022-03729-4 (PMC9528065; doi:10.1186/s12906-022-03729-4)
Supplement: Supplementary file 1 — Additional file 1. [file 12906_2022_3729_MOESM1_ESM.zip › Drug Target.docx]

PGR

PGR

NCOA2

PTGS1

PTGS2

KCNH2

CHRM3

CHRM1

SCN5A

CHRM4

PDE3A

ADRA1A

CHRM2

ADRA1B

ADRB2

CHRNA2

SLC6A4

OPRM1

GABRA1

BCL2

BAX

CASP9

JUN

CASP3

CASP8

PRKCA

PON1

MAP2

NOS2

PTGS1

AR

PTGS2

NCOA2

PRSS1

PGR

CHRM1

ACHE

SLC6A2

CHRM2

ADRA1B

GABRA1

F7

RELA

IKBKB

AKT1

BCL2

BAX

TNF

JUN

AHSA1

CASP3

MAPK8

XDH

MMP1

STAT1

PPARG

HMOX1

CYP3A4

CYP1A2

CYP1A1

ICAM1

SELE

VCAM1

NR1I2

CYP1B1

HAS2

GSTP1

AHR

PSMD3

SLC2A4

NR1I3

INSR

DIO1

PPP3CA

GSTM1

GSTM2

AKR1C3

SLPI

PTGS1

ESR1

PTGS2

NCOA2

RXRA

CAT

HAS2

F7

PTGS1

AR

PTGS2

NCOA2

PRSS1

KCNH2

SCN5A

ADRB2

MMP3

F7

RXRA

ACHE

GABRA1

RELA

EGFR

AKT1

VEGFA

CCND1

BCL2

BCL2L1

FOS

CDKN1A

EIF6

BAX

CASP9

PLAU

MMP2

MMP9

MAPK1

IL10

EGF

RB1

TNF

JUN

IL6

AHSA1

CASP3

TP53

ELK1

NFKBIA

POR

ODC1

XDH

CASP8

TOP1

RAF1

PRKCA

MMP1

HIF1A

STAT1

RUNX1T1

ERBB2

PPARG

ACACA

HMOX1

CYP3A4

CYP1A2

CAV1

MYC

F3

GJA1

CYP1A1

ICAM1

IL1B

CCL2

SELE

VCAM1

PTGER3

CXCL8

PRKCB

BIRC5

DUOX2

NOS3

HSPB1

MGAM

IL2

NR1I2

CYP1B1

CCNB1

PLAT

THBD

SERPINE1

IFNG

IL1A

MPO

TOP2A

NCF1

HAS2

GSTP1

NFE2L2

AHR

PSMD3

SLC2A4

CXCL11

CXCL2

DCAF5

NR1I3

CHEK2

INSR

CLDN4

PPARA

PPARD

HSF1

CRP

CXCL10

CHUK

SPP1

RUNX2

RASSF1

E2F1

E2F2

ACP3

CTSD

IGFBP3

IGF2

CD40LG

IRF1

ERBB3

PON1

DIO1

PCOLCE

NPEPPS

HK2

NKX3-1

RASA1

GSTM1

GSTM2
