# Supplementary material for: Effect and mechanisms of kaempferol against endometriosis based on network pharmacology and in vitro experiments
Source: BMC Complement Med Ther. 2022 Oct 2;22:254. doi: 10.1186/s12906-022-03729-4 (PMC9528065; doi:10.1186/s12906-022-03729-4)
Supplement: Supplementary file 1 — Additional file 1. [file 12906_2022_3729_MOESM1_ESM.zip › EMS GENES.docx]

CYP19A1

IL10

KRAS

PGR

PTGS2

HDAC2

CCL11

GREB1

CDKN2B-AS1

ESR2

NR5A1

HSD17B1

EGFR

IGF1

HSD17B2

PRL

IGFBP1

IL15

IL1R1

MIR21

SST

IDO1

ARNT

PTGER4

KLF9

TGFB2

AKR1C3

ABCC4

FOS

NR3C1

PAPPA

NR2F2

NCOA1

OLFM4

CXCL13

TXNIP

AKR1C1

AKR1C2

AKR1B1

DICER1

HDAC1

HLA-DPB1

NR4A1

TNC

ITGB1

LTF

CXCL9

NR3C2

PAX2

KLF13

ENPP1

PLA2G2A

ACTA2

RXFP1

CCL1

TAGLN

MTA1

MTA2

CXCL14

MED16

ABCC9

UST

RASGRP1

TOB1

PLXNC1

CD226

BRD8

SMPDL3A

DEPP1

OSR2

LRRK2

ANO4

CNR1

CPM

VCAN

CNIH3

CYB5A

CYP26A1

CD55

DDX5

CFD

DIO2

HBEGF

DUSP1

ELAVL1

NR2F6

FBLN1

FBN1

FKBP5

DKK1

NCOA6

FMO2

NEDD4L

ITGB3BP

SLC7A8

LMOD1

METTL7A

ABI3BP

ANKRD1

GPX3

MED4

MYLIP

SLC40A1

IFIT1

IFNGR1

IGFBP6

RBPJ

IHH

IL7R

IMPA2

ITGA2

ITGB8

AREG

RASL11A

FAM180A

LAMB1

TACSTD2

MAOA

MAOB

MMP2

MMP9

NEFM

NTRK3

HERC5

ERRFI1

MED1

SULF2

ANKH

PRLR

PTGER2

PTGFR

RARB

RGS4

RORB

CCL22

SELENOP

SLC1A1

BMP7

SLC20A1

SRD5A1

SRD5A2

THRA

TNF

C1R

NR2C1

NR2C2

TRH

VEGFA

NDNF

ARHGAP28

DCSTAMP

SPARCL1

STC2

SUCLG2

NRP1

CLDN1

SLC16A6

CCNE2

SCGB3A1

MED14

COPS2

MED17

NCOR1

HS3ST3B1

NR1D2

CCL5

BCL2

KDR

BIRC5

SERPINF1

EGR1

RUNX1

HDAC3

ESR1

LYN

NFKBIA

WNT4

BAX

BCL2L1

SELL

VAV1

FN1

VEZT

BSG

IL33

CDC42

IGF1R

SYNE1

MAP3K4

CCDC170

LINC00861

LAMC3

GPNMB

ARID3B

MTCO2P12

CALHM3

COL12A1

COMT

RFLNA

CTNNB1

CYP1A1

CYP2B6

CYP17A1

CYP21A2

AKT1

OR9Q1

NAALADL2

GRIN2D

GSTM1

GSTT1

HOXA10

ICAM1

IL1A

IL1B

IL6

CXCL8

INHBA

MEIS1

MME

CAPN14

COX2

PDE1C

HSD17B7

PIK3CA

PIK3CB

PIK3CD

PIK3CG

FGD6

MAPK1

PTEN

CCL2

TGFB1

TP53

C2

CACNA1A

THOC6

CDC73

BEND5

ARID1A

AGBL4

SKAP1

SYNJ2

FOXP2

MUC16

AGRP

HGF

HIF1A

MIF

SERPINE1

BDNF

CXCL12

CXCR4

PPP1R2C

ARTN

GALT

GSTM2

IFNG

MIR451A

BRD2

STAT3

IL1R2

CDH1

EPHB2

FSHR

AMH

HPGDS

HP

IL4

MALAT1

LEP

STS

MMP1

PAEP

MAPK3

TLR4

BECN1

FGF2

IL37

GPER1

GSTP1

IL18

MMP3

TAC1

CD44

CDKN1B

AHSA1

FCRL3

CCR1

CRH

CRK

MAPK14

CYP2C19

EGF

RNF19A

POLDIP2

PTPN22

ANGPT2

HLA-DRB1

HOXA11

IL1RN

MIRLET7B

MIR145

MIR20A

NOS3

NOTCH1

FOXP3

PPARG

RAF1

S100A6

SPP1

TIMP1

TNFRSF1B

UCN

VCAM1

VDR

AIMP2

KLF11

GRAP2

NAT2

CDKN2A

FST

HPSE

SLCO6A1

CRP

CSF1

DRD2

EZH2

KLRK1

GNRHR

HLA-G

HMGB1

HSD17B13

IL13

IL16

IL17A

GSTK1

MIR17

MIR210

MMP7

MRC1

MUC1

NGF

OGG1

PLAU

POU5F1

AHRR

POU5F1P3

POU5F1P4

BRCA1

BRAF

BRCA2

SULT1E1

HNF1B

TERT

THBS1

TIMP2

XRCC1

DHRS11

PTGES

KLRC4-KLRK1

YAP1

CIB1

POSTN

CFL1

CCR9

H3P10

CREB1

CRHR1

CCN2

CTLA4

CYP1B1

ACE

AGTR1

AHR

F3

FGFR2

FOXO1

SIRT1

MTOR

FSHB

GATA6

GC

GHRH

ANXA1

CCN1

IL1RAP

FASLG

CXCL10

ISG20

AR

ITGAM

ITGAV

LEPR

LGALS3

MIR126

MIR141

SMAD3

MDK

MMP14

MPO

ATM

NFKB1

NME1

TNFRSF11B

PCNA

PGF

PLG

PPARA

VPS11

MAPK8

ACKR3

CCND1

REN

ACTB

RNASE3

CCL25

SOD2

STAR

TACR1

ZEB1

TNFRSF1A

TWIST1

EZR

CD36

CD68

CDH3

SRA1

ABCB6

EMSLR

CDK6

CDKN1A

LINC02210-CRHR1

CEBPA

SEMA3C

KHDRBS1

PPARGC1A

STIP1

CKS1B

CCR5

CKS1BP7

CRHR2

LINC00261

CSF1R

CSF2

CSF3

RMDN2

DNMT3A

DNMT3B

AGT

DUSP2

AGTR2

AHSG

AIF1

EMX2

ENO1

EPO

ERBB2

FGA

FGF1

FKBP4

ZHX2

FOXM1

FLT1

CAPN7

FPR1

FPR2

NUP62

RNU1-1

GH1

GJA1

PDCD4

GLI1

ANG

CXCR3

ANGPT1

LINC00339

CD274

GTF2H1

ANXA2

ERVW-1

HMGA1

HOXA13

APEX1

HSD11B2

HSPA4

IGFBP3

FAS

IL2RA

IL2RB

IL7

CXCR1

CXCR2

ITGB3

KIR2DS1

KIR2DS5

LHCGR

LIF

LPA

LTA

MIR143

MIR183

MIR191

MIR200C

MIR29C

MIR31

SMAD2

MLH1

MIR342

MSH2

MSI1

MST1

MST1R

MUC2

MUC4

NCAM1

NOS1

NOS2

NTF4

NTRK2

OPRM1

P2RX3

SERPINB2

PAK1

IL22

RMDN1

DCTN4

IL23A

ENPP3

ACP1

PON1

KRT20

RMDN3

SYBU

MAP2K7

RETN

SEMA6A

RB1

RELA

BCL6

RNU1-4

S100B

CCL21

CXCL5

SKP2

SLC2A4

SLIT3

SOD1

SOX2

SOX9

SRC

SSTR1

STAT6

STC1

MIR33B

TCF21

THY1

TIMP3

TLR2

C3

CCR2

USF2

VEGFC

VIM

WNT7A

XRCC4

PAX8

MANEA

COL18A1

HMGA2

PROK1

HSH2D

TSLP

LGR5

TNFSF10

NR1I2

SQSTM1

CCNE1

USP10

IL32

MS4A1

PPIG

ADIPOQ

CD47

ADA

MIR543

MR1P1

PDCD6

BCL2L11

DEFB4B

MIR1185-1

TMED7-TICAM2

MIR2861

MIR3613

LINC01541

LINC01672

DNM1L

MIR4634

PCAT1

PGR-AS1

KIF20A

CDH15

HNRNPA3P1

CDK4

TSHZ1

EIF1

LILRB2

PAK4

ADAR

CDKN3

NOD1

NDRG1

MYL9

CDX1

CARM1

ZNRD2

AGR2

PDLIM5

CXCR6

CTCF

DCTN6

TNFSF13B

CETP

CFTR

LILRB1

MMP24

UTS2

LOC110386951

LOC110806263

NUDT6

AKAP13

GALNT6

PRRT2

SCN11A

LINC02605

CHRM3

PHB2

UCN3

CHUK

H3P23

CTHRC1

CLCN3

ANTXR2

CYP2R1

H4-16

CCR7

CCR8

LTB4R

COL1A1

RBM45

SGPP2

CD200R1

ZFP42

ADM

CPB2

CLDN4

CLDN3

CLDN7

CRABP2

DOCK11

CRMP1

MUC17

SIRPA

PARP1

CSE1L

CSK

IL34

ADRA1A

CTH

PDIK1L

ADRA2B

GPBAR1

CCDC80

CX3CR1

CYP1A2

CYP3A4

TTC39B

CYP11A1

DAPK1

DCN

DECR1

DEFB4A

ZNF366

ARX

DLX3

DLX5

SARDH

DNMT1

DOK1

DPP4

DPP6

DPYSL2

DUSP6

S1PR1

EDN1

EDNRA

MEGF8

EIF4EBP1

ELAVL2

TET3

EPHA3

EPHB4

EPOR

ERCC1

ERCC2

ERCC6

ESRRB

ETS1

ALB

EXTL3

F2

F2R

F2RL1

FANCD2

FCGR3A

FCGR3B

HOXA11-AS

FEN1

FGF9

KIFAP3

FOXO3

PLXND1

NUP210

PLCB1

ASTN2

HEY1

SRRM2

LPAR3

FAM215A

DAPK2

SH3BP4

CADM1

FJX1

ALOX15

IL27

ALPI

NR5A2

ALPP

CADM2

GABPA

GALNT3

NCR3

NECTIN3

CHD5

GATA2

GATA3

GATM

GBA

GREM1

MSTN

GDNF

AMHR2

GHRHR

FOXD3

GJB2

FOXP1

CACYBP

INTU

TNFRSF21

RBMS3

GLI3

GNRH1

ANK1

GPR42

GPX4

GRB2

CCDC22

REM1

CXCL1

PDIA3

GSK3B

MSH6

TMOD3

DNMT3L

TBX21

H2AX

HAS1

HCK

HK1

ACACA

HLA-B

HLA-C

HLA-DQB1

FOXA2

HNF4A

HOXB4

HPGD

HPRT1

AGFG2

HSD3B1

HSD3B2

BIRC2

HSD17B3

BIRC3

HSPD1

HTC2

APOA2

IAPP

ID2

CPP

CFI

IFI27

IFNA1

IFNA13

APOE

IGFBP7

TICAM2

IL2

IL9

AQP1

IL11

AQP2

IL12B

IL12RB1

ILK

AQP5

INHA

INSR

EIF3E

AQP9

ITGA5

JAK1

JUN

CD82

USP17L2

KCNQ1

KCNQ2

KIR2DL1

KIR2DL3

KIR2DL4

KIR3DL1

KIR3DL2

KISS1

KLRC1

KNG1

ARG1

TBPL2

HES5

RHOC

L1CAM

RHOG

LAMC2

STMN1

LBR

LGALS1

LGALS4

LGALS9

LHB

LIMK1

LIPC

LIPE

LMNB1

LOX

LOXL1

CYP4F3

SH2D1A

MIRLET7D

MIR100

MIR106A

MIR10B

MIR122

MIR132

MIR139

MIR142

MIR148A

MIR154

MIR181C

MIR182

MIR195

MIR196A2

MIR200A

MIR200B

MIR204

MIR205

MIR214

MIR216A

MIR22

MIR23B

MIR27B

MIR30A

MIR30C1

MIR30C2

MIR33A

MIR34A

MIR34B

EPCAM

SMAD4

MAP2

ARSD

MAS1

MAT2A

MBL2

MDM2

MECP2

MET

KITLG

MMP12

MMP13

MNAT1

CD200

CCDC144NL-AS1

MIR135B

MIR370

MIR196B

MSMB

MYB

MYC

NFE2L2

NGFR

NHS

NINJ1

NOTCH4

NPTX2

NRAS

YBX1

NT5E

NTF3

MIR375

MIR378A

MIR381

CLDN11

OXTR

P2RX5

P2RY6

MBL3P

TMED7

HSPA14

NRN1

PDCD1

VTA1

PDGFA

PDGFRA

PDGFRB

PF4

PF4V1

PHB

SERPINA1

PLCB4

IL17D

PLK1

PNN

TREM2

POLD1

POLE

UGT2B28

RHOF

ROBO4

DLL4

UGT1A1

RBFOX1

BNC2

SLC52A1

PPP2R1A

ATAD3A

VPS53

QRSL1

PPP5C

PPT1

H4C15

PRELP

PRKCA

PRKCB

AXL

ST6GALNAC1

TRERF1

MYDGF

MAP2K1

LTB4R2

HTRA1

PSEN1

B2M

PNO1

PSMD2

PSMD9

PSMD10

SALL4

PTBP1

MIR363

MIR488

MIR146B

MIR520G

MIR503

PTN

PTPRC

PTPRD

PVR

NECTIN1

CXCL16

IL21

RARRES1

RARRES2

RASA1

RBP1

AFAP1

ROCK1

ROS1

MIR483

RXRA

S100A1

S100A4

SAG

SLC22A23

SRL

CCL14

CCL16

CCL17

CCL19

CX3CL1

SDC1

SRR

SDC4

SELE

SMOC2

SFRP1

SFRP2

DCLRE1C

ATG3

SHC1

GORASP1

WNK1

SLC2A1

SLC2A3

BMP4

SLC6A4

BMPR1B

SLPI

SNAI2

SMARCA1

SMARCC1

SNAI1

SNCG

FSCN1

MIR542

SOX15

SRY

SSTR4

STAT4

BRS3

STX5

SULT1A1

SULT2A1

SYP

SYT1

ADAM17

BTF3P11

TCF3

MIR449B

MIR629

BTG1

BTK

TDGF1

TEK

TERF1

TFDP1

TFF3

TGFB1I1

TGFB3

LEFTY2

TGFBI

TGFBR1

TSPO

KLF10

TIMP4

TLR3

SERPING1

CLDN5

TNS1

TPM3

TPT1

TRO

TRPC6

C5

TXN

TYK2

RGPD2

TYROBP

UCHL1

C9

UTRN

VIP

TRPV1

VTN

VWF

WT1

XBP1

XRCC3

YWHAZ

ZFP36

CA2

ZNF217

ZP3

BAG6

ULBP3

CALCA

NAA16

NANOG

STN1

CALD1

RNF34

SPHKAP

TET1

ULBP2

ACTN4

SLC38A1

NECTIN4

CAB39L

MAP1LC3B

MIA

H3-4

H4C9

AXIN1

FZD7

H4C1

CASP3

H4C4

H4C6

H4C12

H4C11

H4C3

H4C8

H4C2

H4C5

H4C13

H4C14

CRISPLD2

TMPRSS13

MAGT1

BCAR3

NLRC5

LOXL4

NR0B2

UTF1

CARD11

MAK16

CASR

KISS1R

CAT

AFAP1-AS1

GFM1

DENR

RUNX3

IRS2

PEA15

HYAL2

ACTN1

SERPINH1

TNFRSF6B

IL18R1

PROM1

IER3

CCNB1

BCL10

WNT3A

LMLN

UCN2

CREB3L1

ACVR1B

EBAG9

LPAR2

SLC33A1

CD14

S1PR2

ACVR2B

CD19

IGSF8

CD163

SLIT2

LIPG

SFXN1

CD86

HAND2

CHST3

TECR

ENTPD1

BCAR1

GDF3

CLOCK

CD40LG

CD48

RGS6

IPO13

CD74

SEMA3E

CD79A

HDAC9

RASSF2

BMS1

CDK1

CDC6
