# Supplementary material for: Effect and mechanisms of kaempferol against endometriosis based on network pharmacology and in vitro experiments
Source: BMC Complement Med Ther. 2022 Oct 2;22:254. doi: 10.1186/s12906-022-03729-4 (PMC9528065; doi:10.1186/s12906-022-03729-4)
Supplement: Supplementary file 1 — Additional file 1. [file 12906_2022_3729_MOESM1_ESM.zip › GO.docx]

| GO:0070851 | growth factor receptor binding | 6月30日 | 134/17697 | 8.61E-08 | 6.06E-06 | 2.81E-06 | IL6/VEGFA/EGF/IL1B/IL10/PTEN | 6 |
| --- | --- | --- | --- | --- | --- | --- | --- | --- |
| GO:0033613 | activating transcription factor binding | 5月30日 | 85/17697 | 2.94E-07 | 1.55E-05 | 7.20E-06 | JUN/MYC/FOS/PPARG/RELA | 5 |
| GO:0048018 | receptor ligand activity | 8月30日 | 482/17697 | 9.87E-07 | 4.16E-05 | 1.93E-05 | IL6/VEGFA/TNF/EGF/CXCL8/IL1B/CCL2/IL10 | 8 |
| GO:0002020 | protease binding | 5月30日 | 128/17697 | 2.26E-06 | 7.93E-05 | 3.68E-05 | TP53/TNF/CASP3/PTEN/SERPINE1 | 5 |
| GO:0001085 | RNA polymerase II transcription factor binding | 5月30日 | 155/17697 | 5.77E-06 | 0.000174 | 8.07E-05 | TP53/JUN/ESR1/FOS/PPARG | 5 |
| GO:0019902 | phosphatase binding | 5月30日 | 185/17697 | 1.36E-05 | 0.00036 | 0.000167 | AKT1/TP53/MAPK1/EGFR/PPARG | 5 |
| GO:0042826 | histone deacetylase binding | 4月30日 | 111/17697 | 3.54E-05 | 0.00083 | 0.000385 | TP53/MAPK8/CCND1/RELA | 4 |
| GO:0020037 | heme binding | 4月30日 | 135/17697 | 7.61E-05 | 0.001541 | 0.000715 | PTGS2/CAT/HMOX1/NOS3 | 4 |
| GO:0001228 | DNA-binding transcription activator activity, RNA polymerase II-specific | 6月30日 | 439/17697 | 8.06E-05 | 0.001541 | 0.000715 | TP53/JUN/MYC/ESR1/FOS/RELA | 6 |
| GO:0019903 | protein phosphatase binding | 4月30日 | 140/17697 | 8.76E-05 | 0.001541 | 0.000715 | AKT1/TP53/EGFR/PPARG | 4 |
| GO:0046906 | tetrapyrrole binding | 4月30日 | 145/17697 | 0.0001 | 0.00163 | 0.000756 | PTGS2/CAT/HMOX1/NOS3 | 4 |
| GO:0001047 | core promoter binding | 3月30日 | 55/17697 | 0.000109 | 0.001638 | 0.00076 | TP53/MYC/FOS | 3 |
| GO:0008083 | growth factor activity | 4月30日 | 163/17697 | 0.000158 | 0.002218 | 0.001029 | IL6/VEGFA/EGF/IL10 | 4 |
| GO:0070491 | repressing transcription factor binding | 3月30日 | 71/17697 | 0.000232 | 0.003065 | 0.001422 | MYC/PPARG/RELA | 3 |
| GO:0004707 | MAP kinase activity | 2/30 | 14/17697 | 0.00025 | 0.003098 | 0.001437 | MAPK8/MAPK1 | 2 |
| GO:0005161 | platelet-derived growth factor receptor binding | 2/30 | 15/17697 | 0.000288 | 0.003373 | 0.001565 | VEGFA/PTEN | 2 |
| GO:0001091 | RNA polymerase II basal transcription factor binding | 2/30 | 20/17697 | 0.000518 | 0.005752 | 0.002668 | TP53/ESR1 | 2 |
| GO:0000980 | RNA polymerase II distal enhancer sequence-specific DNA binding | 3月30日 | 99/17697 | 0.000618 | 0.006518 | 0.003024 | TP53/JUN/RELA | 3 |
| GO:0001223 | transcription coactivator binding | 2/30 | 23/17697 | 0.000687 | 0.006593 | 0.003059 | ESR1/RELA | 2 |
| GO:0070412 | R-SMAD binding | 2/30 | 23/17697 | 0.000687 | 0.006593 | 0.003059 | JUN/FOS | 2 |
| GO:0001158 | enhancer sequence-specific DNA binding | 3月30日 | 119/17697 | 0.001054 | 0.009671 | 0.004487 | TP53/JUN/RELA | 3 |
| GO:0051721 | protein phosphatase 2A binding | 2/30 | 32/17697 | 0.001335 | 0.01135 | 0.005266 | AKT1/TP53 | 2 |
| GO:0031625 | ubiquitin protein ligase binding | 4月30日 | 290/17697 | 0.001383 | 0.01135 | 0.005266 | TP53/JUN/EGFR/RELA | 4 |
| GO:0005178 | integrin binding | 3月30日 | 132/17697 | 0.001421 | 0.01135 | 0.005266 | EGFR/IL1B/ICAM1 | 3 |
| GO:0035326 | enhancer binding | 3月30日 | 133/17697 | 0.001452 | 0.01135 | 0.005266 | TP53/JUN/RELA | 3 |
| GO:0044389 | ubiquitin-like protein ligase binding | 4月30日 | 308/17697 | 0.001725 | 0.013002 | 0.006032 | TP53/JUN/EGFR/RELA | 4 |
| GO:0030331 | estrogen receptor binding | 2/30 | 42/17697 | 0.002293 | 0.015944 | 0.007397 | ESR1/PPARG | 2 |
| GO:0001221 | transcription cofactor binding | 2/30 | 43/17697 | 0.002403 | 0.015944 | 0.007397 | ESR1/RELA | 2 |
| GO:0004712 | protein serine/threonine/tyrosine kinase activity | 2/30 | 43/17697 | 0.002403 | 0.015944 | 0.007397 | AKT1/MAPK1 | 2 |
| GO:0016705 | oxidoreductase activity, acting on paired donors, with incorporation or reduction of molecular oxygen | 3月30日 | 159/17697 | 0.002418 | 0.015944 | 0.007397 | PTGS2/HMOX1/NOS3 | 3 |
| GO:0001046 | core promoter sequence-specific DNA binding | 2/30 | 45/17697 | 0.002628 | 0.016333 | 0.007578 | TP53/FOS | 2 |
| GO:0032813 | tumor necrosis factor receptor superfamily binding | 2/30 | 46/17697 | 0.002745 | 0.016333 | 0.007578 | TNF/CASP3 | 2 |
| GO:0042805 | actinin binding | 2/30 | 46/17697 | 0.002745 | 0.016333 | 0.007578 | PPARG/RELA | 2 |
| GO:0004879 | nuclear receptor activity | 2/30 | 47/17697 | 0.002864 | 0.016333 | 0.007578 | ESR1/PPARG | 2 |
| GO:0098531 | transcription factor activity, direct ligand regulated sequence-specific DNA binding | 2/30 | 47/17697 | 0.002864 | 0.016333 | 0.007578 | ESR1/PPARG | 2 |
| GO:0008009 | chemokine activity | 2/30 | 49/17697 | 0.003109 | 0.016822 | 0.007805 | CXCL8/CCL2 | 2 |
| GO:0016538 | cyclin-dependent protein serine/threonine kinase regulator activity | 2/30 | 49/17697 | 0.003109 | 0.016822 | 0.007805 | CASP3/CCND1 | 2 |
| GO:0070888 | E-box binding | 2/30 | 50/17697 | 0.003235 | 0.017067 | 0.007918 | MYC/PPARG | 2 |
| GO:0019887 | protein kinase regulator activity | 3月30日 | 180/17697 | 0.003433 | 0.017149 | 0.007956 | CASP3/EGF/CCND1 | 3 |
| GO:0004601 | peroxidase activity | 2/30 | 52/17697 | 0.003495 | 0.017149 | 0.007956 | PTGS2/CAT | 2 |
| GO:0050661 | NADP binding | 2/30 | 52/17697 | 0.003495 | 0.017149 | 0.007956 | CAT/NOS3 | 2 |
| GO:0001102 | RNA polymerase II activating transcription factor binding | 2/30 | 53/17697 | 0.003628 | 0.017398 | 0.008072 | JUN/FOS | 2 |
| GO:0003707 | steroid hormone receptor activity | 2/30 | 56/17697 | 0.004042 | 0.018145 | 0.008418 | ESR1/PPARG | 2 |
| GO:0016684 | oxidoreductase activity, acting on peroxide as acceptor | 2/30 | 56/17697 | 0.004042 | 0.018145 | 0.008418 | PTGS2/CAT | 2 |
| GO:0043621 | protein self-association | 2/30 | 56/17697 | 0.004042 | 0.018145 | 0.008418 | TP53/PPARG | 2 |
| GO:0005516 | calmodulin binding | 3月30日 | 200/17697 | 0.004611 | 0.020269 | 0.009404 | AKT1/EGFR/NOS3 | 3 |
| GO:0019207 | kinase regulator activity | 3月30日 | 207/17697 | 0.005074 | 0.021851 | 0.010138 | CASP3/EGF/CCND1 | 3 |
| GO:0042379 | chemokine receptor binding | 2/30 | 66/17697 | 0.005571 | 0.023509 | 0.010907 | CXCL8/CCL2 | 2 |
| GO:0004674 | protein serine/threonine kinase activity | 4月30日 | 439/17697 | 0.006142 | 0.02541 | 0.011789 | AKT1/MAPK8/MAPK1/EGFR | 4 |
| GO:0001098 | basal transcription machinery binding | 2/30 | 72/17697 | 0.006597 | 0.026262 | 0.012184 | TP53/ESR1 | 2 |
| GO:0001099 | basal RNA polymerase II transcription machinery binding | 2/30 | 72/17697 | 0.006597 | 0.026262 | 0.012184 | TP53/ESR1 | 2 |
| GO:0001618 | virus receptor activity | 2/30 | 74/17697 | 0.006956 | 0.026687 | 0.012381 | EGFR/ICAM1 | 2 |
| GO:0104005 | hijacked molecular function | 2/30 | 74/17697 | 0.006956 | 0.026687 | 0.012381 | EGFR/ICAM1 | 2 |
| GO:0001227 | DNA-binding transcription repressor activity, RNA polymerase II-specific | 3月30日 | 242/17697 | 0.007808 | 0.029421 | 0.01365 | MYC/PPARG/RELA | 3 |
| GO:0046332 | SMAD binding | 2/30 | 80/17697 | 0.008087 | 0.029937 | 0.01389 | JUN/FOS | 2 |
| GO:0016209 | antioxidant activity | 2/30 | 86/17697 | 0.009296 | 0.033816 | 0.015689 | PTGS2/CAT | 2 |
| GO:0035258 | steroid hormone receptor binding | 2/30 | 92/17697 | 0.010579 | 0.037834 | 0.017554 | ESR1/PPARG | 2 |
| GO:1990782 | protein tyrosine kinase binding | 2/30 | 93/17697 | 0.0108 | 0.037982 | 0.017622 | TP53/PTEN | 2 |
| GO:0004222 | metalloendopeptidase activity | 2/30 | 103/17697 | 0.013124 | 0.045396 | 0.021062 | MMP9/MMP2 | 2 |
| GO:0047485 | protein N-terminus binding | 2/30 | 109/17697 | 0.014614 | 0.049733 | 0.023074 | TP53/RELA | 2 |
