# Supplementary material for: Effect and mechanisms of kaempferol against endometriosis based on network pharmacology and in vitro experiments
Source: BMC Complement Med Ther. 2022 Oct 2;22:254. doi: 10.1186/s12906-022-03729-4 (PMC9528065; doi:10.1186/s12906-022-03729-4)
Supplement: Supplementary file 1 — Additional file 1. [file 12906_2022_3729_MOESM1_ESM.zip › KEGG.docx]

| hsa04933 | AGE-RAGE signaling pathway in diabetic complications | 100/8108 | 8.97E-26 | 1.79E-23 | 3.87E-24 | AKT1/IL6/VEGFA/TNF/MAPK8/CASP3/JUN/MAPK1/CXCL8/IL1B/CCL2/MMP2/CCND1/SERPINE1/ICAM1/RELA/NOS3 | 17 |
| --- | --- | --- | --- | --- | --- | --- | --- |
| hsa05417 | Lipid and atherosclerosis | 215/8108 | 7.44E-20 | 7.40E-18 | 1.60E-18 | AKT1/IL6/TP53/TNF/MAPK8/CASP3/JUN/MAPK1/MMP9/CXCL8/IL1B/CCL2/FOS/PPARG/ICAM1/RELA/NOS3 | 17 |
| hsa05418 | Fluid shear stress and atherosclerosis | 139/8108 | 1.88E-19 | 1.24E-17 | 2.70E-18 | AKT1/TP53/VEGFA/TNF/MAPK8/JUN/MMP9/IL1B/CCL2/FOS/MMP2/HMOX1/ICAM1/RELA/NOS3 | 15 |
| hsa04668 | TNF signaling pathway | 112/8108 | 4.84E-19 | 2.41E-17 | 5.22E-18 | AKT1/IL6/TNF/MAPK8/CASP3/JUN/MAPK1/PTGS2/MMP9/IL1B/CCL2/FOS/ICAM1/RELA | 14 |
| hsa04657 | IL-17 signaling pathway | 94/8108 | 2.96E-18 | 1.18E-16 | 2.55E-17 | IL6/TNF/MAPK8/CASP3/JUN/MAPK1/PTGS2/MMP9/CXCL8/IL1B/CCL2/FOS/RELA | 13 |
| hsa05142 | Chagas disease | 102/8108 | 9.04E-18 | 3.00E-16 | 6.50E-17 | AKT1/IL6/TNF/MAPK8/JUN/MAPK1/CXCL8/IL1B/CCL2/FOS/IL10/SERPINE1/RELA | 13 |
| hsa05167 | Kaposi sarcoma-associated herpesvirus infection | 194/8108 | 3.18E-17 | 9.03E-16 | 1.96E-16 | AKT1/IL6/TP53/VEGFA/MAPK8/CASP3/JUN/MAPK1/PTGS2/CXCL8/MYC/FOS/CCND1/ICAM1/RELA | 15 |
| hsa05219 | Bladder cancer | 41/8108 | 9.34E-17 | 2.32E-15 | 5.04E-16 | TP53/VEGFA/EGF/MAPK1/EGFR/MMP9/CXCL8/MYC/MMP2/CCND1 | 10 |
| hsa05163 | Human cytomegalovirus infection | 225/8108 | 3.00E-16 | 6.64E-15 | 1.44E-15 | AKT1/IL6/TP53/VEGFA/TNF/CASP3/MAPK1/EGFR/PTGS2/CXCL8/MYC/IL1B/CCL2/CCND1/RELA | 15 |
| hsa05133 | Pertussis | 76/8108 | 1.10E-15 | 2.18E-14 | 4.74E-15 | IL6/TNF/MAPK8/CASP3/JUN/MAPK1/CXCL8/IL1B/FOS/IL10/RELA | 11 |
| hsa05161 | Hepatitis B | 162/8108 | 4.45E-15 | 7.61E-14 | 1.65E-14 | AKT1/IL6/TP53/TNF/MAPK8/CASP3/JUN/MAPK1/MMP9/CXCL8/MYC/FOS/RELA | 13 |
| hsa05210 | Colorectal cancer | 86/8108 | 4.59E-15 | 7.61E-14 | 1.65E-14 | AKT1/TP53/MAPK8/CASP3/EGF/JUN/MAPK1/EGFR/MYC/FOS/CCND1 | 11 |
| hsa01522 | Endocrine resistance | 98/8108 | 2.05E-14 | 3.13E-13 | 6.80E-14 | AKT1/TP53/MAPK8/JUN/MAPK1/EGFR/MMP9/ESR1/FOS/MMP2/CCND1 | 11 |
| hsa05135 | Yersinia infection | 137/8108 | 2.22E-14 | 3.16E-13 | 6.85E-14 | AKT1/IL6/TNF/MAPK8/JUN/MAPK1/CXCL8/IL1B/CCL2/FOS/IL10/RELA | 12 |
| hsa05166 | Human T-cell leukemia virus 1 infection | 219/8108 | 2.28E-13 | 3.03E-12 | 6.56E-13 | AKT1/IL6/TP53/TNF/MAPK8/JUN/MAPK1/MYC/FOS/CCND1/PTEN/ICAM1/RELA | 13 |
| hsa04010 | MAPK signaling pathway | 294/8108 | 4.32E-13 | 5.32E-12 | 1.15E-12 | AKT1/TP53/VEGFA/TNF/MAPK8/CASP3/EGF/JUN/MAPK1/EGFR/MYC/IL1B/FOS/RELA | 14 |
| hsa04926 | Relaxin signaling pathway | 129/8108 | 4.54E-13 | 5.32E-12 | 1.15E-12 | AKT1/VEGFA/MAPK8/JUN/MAPK1/EGFR/MMP9/FOS/MMP2/RELA/NOS3 | 11 |
| hsa04620 | Toll-like receptor signaling pathway | 104/8108 | 1.88E-12 | 1.93E-11 | 4.19E-12 | AKT1/IL6/TNF/MAPK8/JUN/MAPK1/CXCL8/IL1B/FOS/RELA | 10 |
| hsa04625 | C-type lectin receptor signaling pathway | 104/8108 | 1.88E-12 | 1.93E-11 | 4.19E-12 | AKT1/IL6/TNF/MAPK8/JUN/MAPK1/PTGS2/IL1B/IL10/RELA | 10 |
| hsa05224 | Breast cancer | 147/8108 | 1.94E-12 | 1.93E-11 | 4.19E-12 | AKT1/TP53/EGF/JUN/MAPK1/EGFR/MYC/ESR1/FOS/CCND1/PTEN | 11 |
| hsa05205 | Proteoglycans in cancer | 205/8108 | 2.87E-12 | 2.72E-11 | 5.90E-12 | AKT1/TP53/VEGFA/TNF/CASP3/MAPK1/EGFR/MMP9/MYC/ESR1/MMP2/CCND1 | 12 |
| hsa04066 | HIF-1 signaling pathway | 109/8108 | 3.04E-12 | 2.75E-11 | 5.97E-12 | AKT1/IL6/VEGFA/EGF/MAPK1/EGFR/SERPINE1/HMOX1/RELA/NOS3 | 10 |
| hsa04932 | Non-alcoholic fatty liver disease | 155/8108 | 3.49E-12 | 3.02E-11 | 6.55E-12 | AKT1/IL6/TNF/MAPK8/CASP3/JUN/CXCL8/IL1B/FOS/PPARG/RELA | 11 |
| hsa05212 | Pancreatic cancer | 76/8108 | 4.19E-12 | 3.48E-11 | 7.54E-12 | AKT1/TP53/VEGFA/MAPK8/EGF/MAPK1/EGFR/CCND1/RELA | 9 |
| hsa04068 | FoxO signaling pathway | 131/8108 | 1.96E-11 | 1.56E-10 | 3.38E-11 | AKT1/IL6/MAPK8/EGF/MAPK1/EGFR/CAT/IL10/CCND1/PTEN | 10 |
| hsa05213 | Endometrial cancer | 58/8108 | 2.15E-11 | 1.65E-10 | 3.57E-11 | AKT1/TP53/EGF/MAPK1/EGFR/MYC/CCND1/PTEN | 8 |
| hsa05323 | Rheumatoid arthritis | 93/8108 | 2.72E-11 | 2.00E-10 | 4.35E-11 | IL6/VEGFA/TNF/JUN/CXCL8/IL1B/CCL2/FOS/ICAM1 | 9 |
| hsa05132 | Salmonella infection | 249/8108 | 2.86E-11 | 2.04E-10 | 4.41E-11 | AKT1/IL6/TNF/MAPK8/CASP3/JUN/MAPK1/CXCL8/MYC/IL1B/FOS/RELA | 12 |
| hsa05162 | Measles | 139/8108 | 3.55E-11 | 2.44E-10 | 5.29E-11 | AKT1/IL6/TP53/MAPK8/CASP3/JUN/IL1B/FOS/CCND1/RELA | 10 |
| hsa05215 | Prostate cancer | 97/8108 | 4.00E-11 | 2.66E-10 | 5.76E-11 | AKT1/TP53/EGF/MAPK1/EGFR/MMP9/CCND1/PTEN/RELA | 9 |
| hsa05169 | Epstein-Barr virus infection | 202/8108 | 6.32E-11 | 4.06E-10 | 8.79E-11 | AKT1/IL6/TP53/TNF/MAPK8/CASP3/JUN/MYC/CCND1/ICAM1/RELA | 11 |
| hsa05207 | Chemical carcinogenesis - receptor activation | 212/8108 | 1.07E-10 | 6.63E-10 | 1.44E-10 | AKT1/VEGFA/EGF/JUN/MAPK1/EGFR/MYC/ESR1/FOS/CCND1/RELA | 11 |
| hsa04218 | Cellular senescence | 156/8108 | 1.12E-10 | 6.78E-10 | 1.47E-10 | AKT1/IL6/TP53/MAPK1/CXCL8/MYC/CCND1/PTEN/SERPINE1/RELA | 10 |
| hsa05160 | Hepatitis C | 157/8108 | 1.20E-10 | 7.01E-10 | 1.52E-10 | AKT1/TP53/TNF/CASP3/EGF/MAPK1/EGFR/MYC/CCND1/RELA | 10 |
| hsa05140 | Leishmaniasis | 77/8108 | 2.26E-10 | 1.28E-09 | 2.78E-10 | TNF/JUN/MAPK1/PTGS2/IL1B/FOS/IL10/RELA | 8 |
| hsa05171 | Coronavirus disease - COVID-19 | 232/8108 | 2.81E-10 | 1.56E-09 | 3.37E-10 | IL6/TNF/MAPK8/JUN/MAPK1/EGFR/CXCL8/IL1B/CCL2/FOS/RELA | 11 |
| hsa05164 | Influenza A | 172/8108 | 2.96E-10 | 1.59E-09 | 3.45E-10 | AKT1/IL6/TNF/CASP3/MAPK1/CXCL8/IL1B/CCL2/ICAM1/RELA | 10 |
| hsa05144 | Malaria | 50/8108 | 4.01E-10 | 2.10E-09 | 4.55E-10 | IL6/TNF/CXCL8/IL1B/CCL2/IL10/ICAM1 | 7 |
| hsa04380 | Osteoclast differentiation | 128/8108 | 4.96E-10 | 2.53E-09 | 5.49E-10 | AKT1/TNF/MAPK8/JUN/MAPK1/IL1B/FOS/PPARG/RELA | 9 |
| hsa05235 | PD-L1 expression and PD-1 checkpoint pathway in cancer | 89/8108 | 7.36E-10 | 3.66E-09 | 7.94E-10 | AKT1/EGF/JUN/MAPK1/EGFR/FOS/PTEN/RELA | 8 |
| hsa05165 | Human papillomavirus infection | 331/8108 | 7.84E-10 | 3.81E-09 | 8.26E-10 | AKT1/TP53/VEGFA/TNF/CASP3/EGF/MAPK1/EGFR/PTGS2/CCND1/PTEN/RELA | 12 |
| hsa04210 | Apoptosis | 136/8108 | 8.55E-10 | 4.05E-09 | 8.79E-10 | AKT1/TP53/TNF/MAPK8/CASP3/JUN/MAPK1/FOS/RELA | 9 |
| hsa05222 | Small cell lung cancer | 92/8108 | 9.63E-10 | 4.41E-09 | 9.56E-10 | AKT1/TP53/CASP3/PTGS2/MYC/CCND1/PTEN/RELA | 8 |
| hsa04915 | Estrogen signaling pathway | 138/8108 | 9.75E-10 | 4.41E-09 | 9.56E-10 | AKT1/JUN/MAPK1/EGFR/MMP9/ESR1/FOS/MMP2/NOS3 | 9 |
| hsa05130 | Pathogenic Escherichia coli infection | 197/8108 | 1.12E-09 | 4.97E-09 | 1.08E-09 | IL6/TNF/MAPK8/CASP3/JUN/MAPK1/CXCL8/IL1B/FOS/RELA | 10 |
| hsa04151 | PI3K-Akt signaling pathway | 354/8108 | 1.69E-09 | 7.33E-09 | 1.59E-09 | AKT1/IL6/TP53/VEGFA/EGF/MAPK1/EGFR/MYC/CCND1/PTEN/RELA/NOS3 | 12 |
| hsa04660 | T cell receptor signaling pathway | 104/8108 | 2.59E-09 | 1.10E-08 | 2.38E-09 | AKT1/TNF/MAPK8/JUN/MAPK1/FOS/IL10/RELA | 8 |
| hsa04917 | Prolactin signaling pathway | 70/8108 | 4.58E-09 | 1.90E-08 | 4.11E-09 | AKT1/MAPK8/MAPK1/ESR1/FOS/CCND1/RELA | 7 |
| hsa05218 | Melanoma | 72/8108 | 5.59E-09 | 2.27E-08 | 4.93E-09 | AKT1/TP53/EGF/MAPK1/EGFR/CCND1/PTEN | 7 |
| hsa05206 | MicroRNAs in cancer | 310/8108 | 6.10E-09 | 2.43E-08 | 5.27E-09 | TP53/VEGFA/CASP3/MAPK1/EGFR/PTGS2/MMP9/MYC/CCND1/PTEN/HMOX1 | 11 |
| hsa05214 | Glioma | 75/8108 | 7.48E-09 | 2.91E-08 | 6.30E-09 | AKT1/TP53/EGF/MAPK1/EGFR/CCND1/PTEN | 7 |
| hsa04071 | Sphingolipid signaling pathway | 119/8108 | 7.59E-09 | 2.91E-08 | 6.30E-09 | AKT1/TP53/TNF/MAPK8/MAPK1/PTEN/RELA/NOS3 | 8 |
| hsa05131 | Shigellosis | 247/8108 | 1.01E-08 | 3.79E-08 | 8.21E-09 | AKT1/TP53/TNF/MAPK8/JUN/MAPK1/EGFR/CXCL8/IL1B/RELA | 10 |
| hsa05152 | Tuberculosis | 180/8108 | 1.03E-08 | 3.79E-08 | 8.21E-09 | AKT1/IL6/TNF/MAPK8/CASP3/MAPK1/IL1B/IL10/RELA | 9 |
| hsa01521 | EGFR tyrosine kinase inhibitor resistance | 79/8108 | 1.08E-08 | 3.91E-08 | 8.49E-09 | AKT1/IL6/VEGFA/EGF/MAPK1/EGFR/PTEN | 7 |
| hsa04621 | NOD-like receptor signaling pathway | 185/8108 | 1.31E-08 | 4.64E-08 | 1.01E-08 | IL6/TNF/MAPK8/JUN/MAPK1/CXCL8/IL1B/CCL2/RELA | 9 |
| hsa04012 | ErbB signaling pathway | 85/8108 | 1.81E-08 | 6.34E-08 | 1.37E-08 | AKT1/MAPK8/EGF/JUN/MAPK1/EGFR/MYC | 7 |
| hsa04510 | Focal adhesion | 201/8108 | 2.70E-08 | 9.25E-08 | 2.01E-08 | AKT1/VEGFA/MAPK8/EGF/JUN/MAPK1/EGFR/CCND1/PTEN | 9 |
| hsa05134 | Legionellosis | 57/8108 | 4.80E-08 | 1.62E-07 | 3.51E-08 | IL6/TNF/CASP3/CXCL8/IL1B/RELA | 6 |
| hsa05231 | Choline metabolism in cancer | 98/8108 | 4.92E-08 | 1.63E-07 | 3.54E-08 | AKT1/MAPK8/EGF/JUN/MAPK1/EGFR/FOS | 7 |
| hsa05146 | Amoebiasis | 102/8108 | 6.51E-08 | 2.12E-07 | 4.60E-08 | IL6/TNF/CASP3/CXCL8/IL1B/IL10/RELA | 7 |
| hsa04659 | Th17 cell differentiation | 107/8108 | 9.07E-08 | 2.91E-07 | 6.32E-08 | IL6/MAPK8/JUN/MAPK1/IL1B/FOS/RELA | 7 |
| hsa04931 | Insulin resistance | 108/8108 | 9.68E-08 | 3.06E-07 | 6.63E-08 | AKT1/IL6/TNF/MAPK8/PTEN/RELA/NOS3 | 7 |
| hsa05321 | Inflammatory bowel disease | 65/8108 | 1.07E-07 | 3.33E-07 | 7.23E-08 | IL6/TNF/JUN/IL1B/IL10/RELA | 6 |
| hsa05225 | Hepatocellular carcinoma | 168/8108 | 1.14E-07 | 3.50E-07 | 7.58E-08 | AKT1/TP53/MAPK1/EGFR/MYC/CCND1/PTEN/HMOX1 | 8 |
| hsa05145 | Toxoplasmosis | 112/8108 | 1.24E-07 | 3.75E-07 | 8.14E-08 | AKT1/TNF/MAPK8/CASP3/MAPK1/IL10/RELA | 7 |
| hsa05120 | Epithelial cell signaling in Helicobacter pylori infection | 70/8108 | 1.68E-07 | 4.91E-07 | 1.07E-07 | MAPK8/CASP3/JUN/EGFR/CXCL8/RELA | 6 |
| hsa05230 | Central carbon metabolism in cancer | 70/8108 | 1.68E-07 | 4.91E-07 | 1.07E-07 | AKT1/TP53/MAPK1/EGFR/MYC/PTEN | 6 |
| hsa05143 | African trypanosomiasis | 37/8108 | 1.96E-07 | 5.58E-07 | 1.21E-07 | IL6/TNF/IL1B/IL10/ICAM1 | 5 |
| hsa05216 | Thyroid cancer | 37/8108 | 1.96E-07 | 5.58E-07 | 1.21E-07 | TP53/MAPK1/MYC/CCND1/PPARG | 5 |
| hsa05223 | Non-small cell lung cancer | 72/8108 | 1.99E-07 | 5.58E-07 | 1.21E-07 | AKT1/TP53/EGF/MAPK1/EGFR/CCND1 | 6 |
| hsa05220 | Chronic myeloid leukemia | 76/8108 | 2.76E-07 | 7.62E-07 | 1.65E-07 | AKT1/TP53/MAPK1/MYC/CCND1/RELA | 6 |
| hsa05170 | Human immunodeficiency virus 1 infection | 212/8108 | 6.83E-07 | 1.86E-06 | 4.04E-07 | AKT1/TNF/MAPK8/CASP3/JUN/MAPK1/FOS/RELA | 8 |
| hsa05226 | Gastric cancer | 149/8108 | 8.78E-07 | 2.36E-06 | 5.12E-07 | AKT1/TP53/EGF/MAPK1/EGFR/MYC/CCND1 | 7 |
| hsa04921 | Oxytocin signaling pathway | 154/8108 | 1.10E-06 | 2.91E-06 | 6.32E-07 | JUN/MAPK1/EGFR/PTGS2/FOS/CCND1/NOS3 | 7 |
| hsa04630 | JAK-STAT signaling pathway | 162/8108 | 1.54E-06 | 4.04E-06 | 8.77E-07 | AKT1/IL6/EGF/EGFR/MYC/IL10/CCND1 | 7 |
| hsa04064 | NF-kappa B signaling pathway | 104/8108 | 1.78E-06 | 4.61E-06 | 9.99E-07 | TNF/PTGS2/CXCL8/IL1B/ICAM1/RELA | 6 |
| hsa04370 | VEGF signaling pathway | 59/8108 | 2.13E-06 | 5.43E-06 | 1.18E-06 | AKT1/VEGFA/MAPK1/PTGS2/NOS3 | 5 |
| hsa04722 | Neurotrophin signaling pathway | 119/8108 | 3.92E-06 | 9.88E-06 | 2.14E-06 | AKT1/TP53/MAPK8/JUN/MAPK1/RELA | 6 |
| hsa05221 | Acute myeloid leukemia | 67/8108 | 4.02E-06 | 1.00E-05 | 2.17E-06 | AKT1/MAPK1/MYC/CCND1/RELA | 5 |
| hsa04919 | Thyroid hormone signaling pathway | 121/8108 | 4.32E-06 | 1.06E-05 | 2.30E-06 | AKT1/TP53/MAPK1/MYC/ESR1/CCND1 | 6 |
| hsa01523 | Antifolate resistance | 31/8108 | 4.47E-06 | 1.09E-05 | 2.35E-06 | IL6/TNF/IL1B/RELA | 4 |
| hsa05010 | Alzheimer disease | 369/8108 | 4.64E-06 | 1.11E-05 | 2.41E-06 | AKT1/IL6/TNF/MAPK8/CASP3/MAPK1/PTGS2/IL1B/RELA | 9 |
| hsa05202 | Transcriptional misregulation in cancer | 192/8108 | 4.80E-06 | 1.14E-05 | 2.47E-06 | IL6/TP53/MMP9/CXCL8/MYC/PPARG/RELA | 7 |
| hsa04115 | p53 signaling pathway | 73/8108 | 6.16E-06 | 1.44E-05 | 3.13E-06 | TP53/CASP3/CCND1/PTEN/SERPINE1 | 5 |
| hsa05415 | Diabetic cardiomyopathy | 203/8108 | 6.94E-06 | 1.61E-05 | 3.48E-06 | AKT1/MAPK8/MMP9/MMP2/PTEN/RELA/NOS3 | 7 |
| hsa04662 | B cell receptor signaling pathway | 82/8108 | 1.09E-05 | 2.50E-05 | 5.42E-06 | AKT1/JUN/MAPK1/FOS/RELA | 5 |
| hsa04211 | Longevity regulating pathway | 89/8108 | 1.63E-05 | 3.69E-05 | 8.01E-06 | AKT1/TP53/CAT/PPARG/RELA | 5 |
| hsa04014 | Ras signaling pathway | 232/8108 | 1.66E-05 | 3.72E-05 | 8.07E-06 | AKT1/VEGFA/MAPK8/EGF/MAPK1/EGFR/RELA | 7 |
| hsa04658 | Th1 and Th2 cell differentiation | 92/8108 | 1.92E-05 | 4.25E-05 | 9.21E-06 | MAPK8/JUN/MAPK1/FOS/RELA | 5 |
| hsa04912 | GnRH signaling pathway | 93/8108 | 2.02E-05 | 4.43E-05 | 9.60E-06 | MAPK8/JUN/MAPK1/EGFR/MMP2 | 5 |
| hsa04061 | Viral protein interaction with cytokine and cytokine receptor | 100/8108 | 2.88E-05 | 6.23E-05 | 1.35E-05 | IL6/TNF/CXCL8/CCL2/IL10 | 5 |
| hsa05022 | Pathways of neurodegeneration - multiple diseases | 476/8108 | 3.62E-05 | 7.75E-05 | 1.68E-05 | IL6/TNF/MAPK8/CASP3/MAPK1/PTGS2/IL1B/CAT/RELA | 9 |
| hsa05203 | Viral carcinogenesis | 204/8108 | 8.45E-05 | 0.000179 | 3.88E-05 | TP53/CASP3/JUN/MAPK1/CCND1/RELA | 6 |
| hsa04664 | Fc epsilon RI signaling pathway | 68/8108 | 0.000105 | 0.00022 | 4.78E-05 | AKT1/TNF/MAPK8/MAPK1 | 4 |
| hsa04920 | Adipocytokine signaling pathway | 69/8108 | 0.000111 | 0.000229 | 4.96E-05 | AKT1/TNF/MAPK8/RELA | 4 |
| hsa05211 | Renal cell carcinoma | 69/8108 | 0.000111 | 0.000229 | 4.96E-05 | AKT1/VEGFA/JUN/MAPK1 | 4 |
| hsa04622 | RIG-I-like receptor signaling pathway | 70/8108 | 0.000118 | 0.000239 | 5.19E-05 | TNF/MAPK8/CXCL8/RELA | 4 |
| hsa04024 | cAMP signaling pathway | 219/8108 | 0.000125 | 0.000251 | 5.45E-05 | AKT1/MAPK8/JUN/MAPK1/FOS/RELA | 6 |
| hsa04137 | Mitophagy - animal | 72/8108 | 0.000132 | 0.000262 | 5.68E-05 | TP53/MAPK8/JUN/RELA | 4 |
| hsa04371 | Apelin signaling pathway | 138/8108 | 0.000134 | 0.000265 | 5.74E-05 | AKT1/MAPK1/CCND1/SERPINE1/NOS3 | 5 |
| hsa01524 | Platinum drug resistance | 73/8108 | 0.000139 | 0.000271 | 5.87E-05 | AKT1/TP53/CASP3/MAPK1 | 4 |
| hsa04072 | Phospholipase D signaling pathway | 148/8108 | 0.000187 | 0.000361 | 7.82E-05 | AKT1/EGF/MAPK1/EGFR/CXCL8 | 5 |
| hsa04310 | Wnt signaling pathway | 166/8108 | 0.000319 | 0.00061 | 0.000132 | TP53/MAPK8/JUN/MYC/CCND1 | 5 |
| hsa05168 | Herpes simplex virus 1 infection | 498/8108 | 0.000336 | 0.000637 | 0.000138 | AKT1/IL6/TP53/TNF/CASP3/IL1B/CCL2/RELA | 8 |
| hsa05020 | Prion disease | 273/8108 | 0.000414 | 0.000777 | 0.000169 | IL6/TNF/MAPK8/CASP3/MAPK1/IL1B | 6 |
| hsa05332 | Graft-versus-host disease | 42/8108 | 0.000476 | 0.000886 | 0.000192 | IL6/TNF/IL1B | 3 |
| hsa04062 | Chemokine signaling pathway | 192/8108 | 0.000622 | 0.001132 | 0.000245 | AKT1/MAPK1/CXCL8/CCL2/RELA | 5 |
| hsa04930 | Type II diabetes mellitus | 46/8108 | 0.000623 | 0.001132 | 0.000245 | TNF/MAPK8/MAPK1 | 3 |
| hsa04060 | Cytokine-cytokine receptor interaction | 295/8108 | 0.000625 | 0.001132 | 0.000245 | IL6/TNF/CXCL8/IL1B/CCL2/IL10 | 6 |
| hsa04935 | Growth hormone synthesis, secretion and action | 119/8108 | 0.0009 | 0.001614 | 0.00035 | AKT1/MAPK8/MAPK1/FOS | 4 |
| hsa04015 | Rap1 signaling pathway | 210/8108 | 0.000934 | 0.001659 | 0.00036 | AKT1/VEGFA/EGF/MAPK1/EGFR | 5 |
| hsa04650 | Natural killer cell mediated cytotoxicity | 131/8108 | 0.001288 | 0.002268 | 0.000492 | TNF/CASP3/MAPK1/ICAM1 | 4 |
| hsa05416 | Viral myocarditis | 60/8108 | 0.001357 | 0.002369 | 0.000514 | CASP3/CCND1/ICAM1 | 3 |
| hsa04623 | Cytosolic DNA-sensing pathway | 63/8108 | 0.001563 | 0.002705 | 0.000587 | IL6/IL1B/RELA | 3 |
| hsa04140 | Autophagy - animal | 141/8108 | 0.00169 | 0.002899 | 0.000629 | AKT1/MAPK8/MAPK1/PTEN | 4 |
| hsa04150 | mTOR signaling pathway | 155/8108 | 0.00239 | 0.004066 | 0.000882 | AKT1/TNF/MAPK1/PTEN | 4 |
| hsa04540 | Gap junction | 88/8108 | 0.004059 | 0.006845 | 0.001484 | EGF/MAPK1/EGFR | 3 |
| hsa04350 | TGF-beta signaling pathway | 94/8108 | 0.004884 | 0.008168 | 0.001771 | TNF/MAPK1/MYC | 3 |
| hsa04640 | Hematopoietic cell lineage | 99/8108 | 0.005645 | 0.009361 | 0.00203 | IL6/TNF/IL1B | 3 |
| hsa05310 | Asthma | 31/8108 | 0.005758 | 0.009469 | 0.002054 | TNF/IL10 | 2 |
| hsa04914 | Progesterone-mediated oocyte maturation | 100/8108 | 0.005805 | 0.009469 | 0.002054 | AKT1/MAPK8/MAPK1 | 3 |
| hsa04215 | Apoptosis - multiple species | 32/8108 | 0.006127 | 0.009913 | 0.00215 | MAPK8/CASP3 | 2 |
| hsa04928 | Parathyroid hormone synthesis, secretion and action | 106/8108 | 0.006823 | 0.01095 | 0.002375 | MAPK1/EGFR/FOS | 3 |
| hsa04725 | Cholinergic synapse | 113/8108 | 0.008138 | 0.012956 | 0.00281 | AKT1/MAPK1/FOS | 3 |
| hsa04670 | Leukocyte transendothelial migration | 114/8108 | 0.008338 | 0.013168 | 0.002856 | MMP9/MMP2/ICAM1 | 3 |
| hsa04726 | Serotonergic synapse | 115/8108 | 0.00854 | 0.013318 | 0.002888 | CASP3/MAPK1/PTGS2 | 3 |
| hsa05330 | Allograft rejection | 38/8108 | 0.008566 | 0.013318 | 0.002888 | TNF/IL10 | 2 |
| hsa04152 | AMPK signaling pathway | 120/8108 | 0.009594 | 0.0148 | 0.00321 | AKT1/CCND1/PPARG | 3 |
| hsa04216 | Ferroptosis | 41/8108 | 0.009923 | 0.01519 | 0.003294 | TP53/HMOX1 | 2 |
| hsa04110 | Cell cycle | 124/8108 | 0.01049 | 0.015814 | 0.00343 | TP53/MYC/CCND1 | 3 |
| hsa04611 | Platelet activation | 124/8108 | 0.01049 | 0.015814 | 0.00343 | AKT1/MAPK1/NOS3 | 3 |
| hsa04940 | Type I diabetes mellitus | 43/8108 | 0.010878 | 0.016276 | 0.00353 | TNF/IL1B | 2 |
| hsa04020 | Calcium signaling pathway | 240/8108 | 0.011205 | 0.01664 | 0.003609 | VEGFA/EGF/EGFR/NOS3 | 4 |
| hsa04728 | Dopaminergic synapse | 132/8108 | 0.012424 | 0.018314 | 0.003972 | AKT1/MAPK8/FOS | 3 |
| hsa04910 | Insulin signaling pathway | 137/8108 | 0.013731 | 0.020091 | 0.004357 | AKT1/MAPK8/MAPK1 | 3 |
| hsa04672 | Intestinal immune network for IgA production | 49/8108 | 0.013974 | 0.02015 | 0.00437 | IL6/IL10 | 2 |
| hsa05030 | Cocaine addiction | 49/8108 | 0.013974 | 0.02015 | 0.00437 | JUN/RELA | 2 |
| hsa04550 | Signaling pathways regulating pluripotency of stem cells | 143/8108 | 0.015399 | 0.022046 | 0.004781 | AKT1/MAPK1/MYC | 3 |
| hsa04723 | Retrograde endocannabinoid signaling | 148/8108 | 0.016874 | 0.023985 | 0.005202 | MAPK8/MAPK1/PTGS2 | 3 |
| hsa04923 | Regulation of lipolysis in adipocytes | 57/8108 | 0.018621 | 0.026281 | 0.0057 | AKT1/PTGS2 | 2 |
| hsa04934 | Cushing syndrome | 155/8108 | 0.019068 | 0.026722 | 0.005795 | MAPK1/EGFR/CCND1 | 3 |
| hsa04390 | Hippo signaling pathway | 157/8108 | 0.019723 | 0.027447 | 0.005952 | MYC/CCND1/SERPINE1 | 3 |
| hsa04217 | Necroptosis | 159/8108 | 0.02039 | 0.028178 | 0.006111 | TNF/MAPK8/IL1B | 3 |
| hsa04213 | Longevity regulating pathway - multiple species | 62/8108 | 0.021813 | 0.029937 | 0.006492 | AKT1/CAT | 2 |
| hsa04929 | GnRH secretion | 64/8108 | 0.023149 | 0.031386 | 0.006807 | AKT1/MAPK1 | 2 |
| hsa04022 | cGMP-PKG signaling pathway | 167/8108 | 0.023185 | 0.031386 | 0.006807 | AKT1/MAPK1/NOS3 | 3 |
| hsa04530 | Tight junction | 169/8108 | 0.023914 | 0.032155 | 0.006974 | MAPK8/JUN/CCND1 | 3 |
| hsa05016 | Huntington disease | 306/8108 | 0.025148 | 0.033587 | 0.007284 | TP53/MAPK8/CASP3/PPARG | 4 |
| hsa05031 | Amphetamine addiction | 69/8108 | 0.026634 | 0.035334 | 0.007663 | JUN/FOS | 2 |
| hsa04520 | Adherens junction | 71/8108 | 0.028084 | 0.037011 | 0.008027 | MAPK1/EGFR | 2 |
| hsa04613 | Neutrophil extracellular trap formation | 190/8108 | 0.032332 | 0.04233 | 0.00918 | AKT1/MAPK1/RELA | 3 |
