# Supplementary material for: Effect and mechanisms of kaempferol against endometriosis based on network pharmacology and in vitro experiments
Source: BMC Complement Med Ther. 2022 Oct 2;22:254. doi: 10.1186/s12906-022-03729-4 (PMC9528065; doi:10.1186/s12906-022-03729-4)
Supplement: Supplementary file 1 — Additional file 1. [file 12906_2022_3729_MOESM1_ESM.zip › WB(3).docx]

GAP


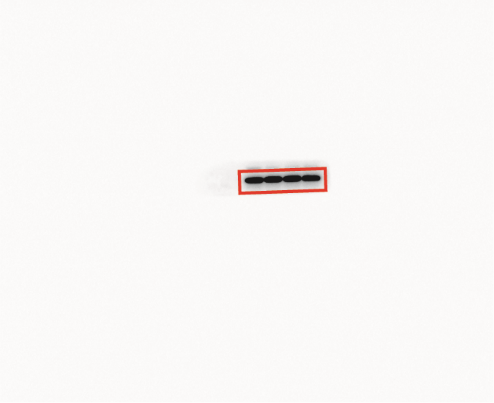

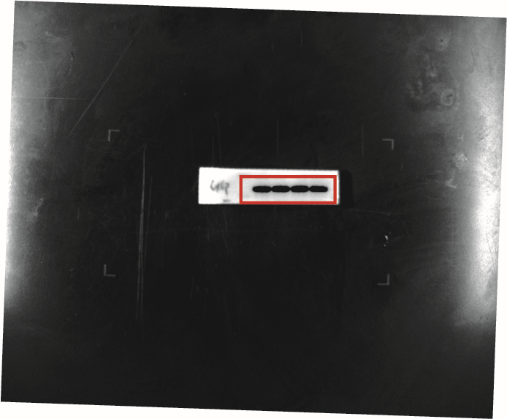


GAP2




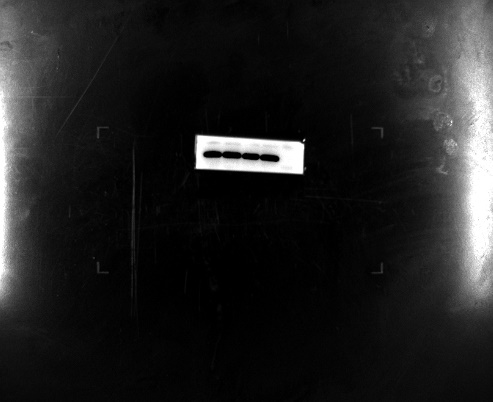


PTEN


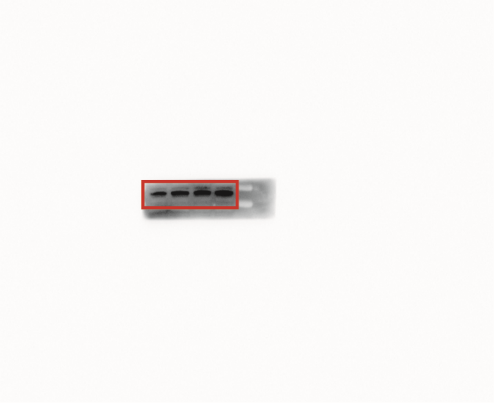

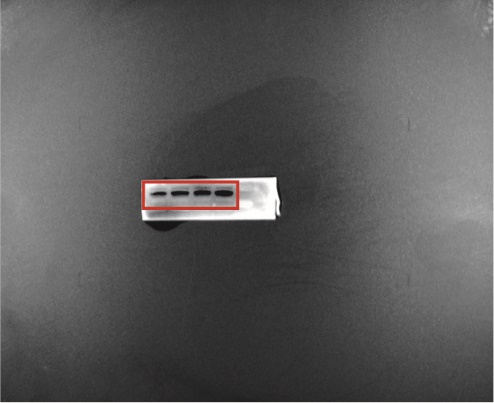


PTEN2




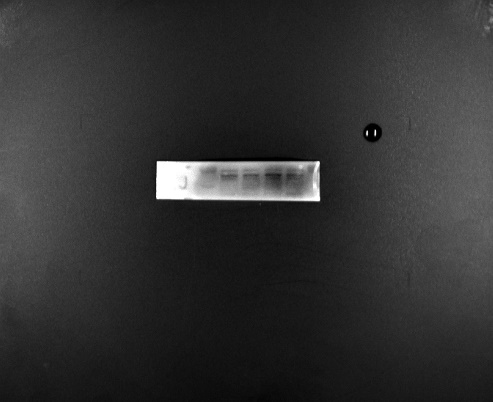


PTEN3




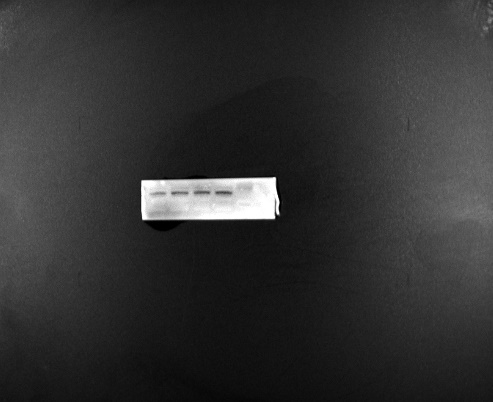


Through our experiments, the expression position of PTEN was about 40-55kD.  Just like the picture we provided, we used a 15-well comb for sample loading, separated by markers in the middle, and the left and right samples were carried out in the same order. In our original data, PTEN1 and PTEN2 were both sides of the same membrane.  Both sides and middle markers were 55kD and 40kD. And the picture of full length membranes showed in the related files.
